# Postbiotics and Paraprobiotics as Next-Generation Gut Microbiome Modulators in Sustainable Aquaculture Health

**DOI:** 10.3390/antibiotics15090887

**Published:** 2026-09-09

**Authors:** Nguyen Vu Linh, Luu Tang Phuc Khang, Patima Permpoonpattana, Nguyen Dinh-Hung

**Affiliations:** 1 Aquatic Biotechnology Laboratory, Department of Animal and Aquatic Sciences, Faculty of Agriculture, Chiang Mai University, Chiang Mai 50200, Thailand; linhvu.n@cmu.ac.th (N.V.L.); khang_luu@cmu.ac.th (L.T.P.K.); 2 Department of Agricultural Science and Technology, Faculty of Innovative Agriculture, Fisheries and Food, Prince of Songkla University, Surat Thani Campus, Surat Thani 84000, Thailand; 3 Aquaculture Pathology Laboratory, School of Animal & Comparative Biomedical Sciences, The University of Arizona, Tucson, AZ 85721, USA

**Keywords:** postbiotics, paraprobiotics, innate immune priming, antibiotic alternatives, gut microbiome, aquafeed

## Abstract

Widespread antibiotic use in aquaculture has increased selective pressure on resident bacterial communities, accelerating the emergence of resistance in key pathogens and raising concerns for animal health, environmental microbiomes, and food-chain safety. Reducing dependence on therapeutic antimicrobials requires alternative strategies that remain effective under the processing and biosafety constraints of intensive production systems, where recurrent bacterial diseases continue to cause substantial economic losses. Live probiotics, currently the most extensively studied microbiome-based intervention, have practical limitations, including reduced viability during feed pelleting and extrusion, transient gut colonization, strain-specific host responses, biosafety concerns related to horizontal transfer of antimicrobial-resistance genes, and variable regulatory requirements across regions. Postbiotics, defined as preparations of non-viable microbial biomass, with or without metabolites, that confer a demonstrated health benefit in the target host, and paraprobiotics, which emphasize inactivated whole-cell preparations that preserve surface-associated microbial molecular patterns, may help address several of these constraints. These approaches offer improved compositional definition, greater feed-processing stability, and a potentially more favorable biosafety profile. Because they are non-viable, their anti-pathogen effects do not depend on growth or competitive colonization but may instead involve preformed antimicrobial compounds retained in some preparations, interference with pathogen attachment, modulation of the intestinal environment, reinforcement of barrier function, and stimulation of host immune responses. This review synthesizes current evidence on postbiotics and paraprobiotics in aquaculture, with emphasis on structural classification, pattern-recognition receptor signaling, intestinal barrier function, innate immune priming, encapsulation technologies, and translational readiness. Taken together, available evidence supports postbiotics and paraprobiotics as promising, but not yet fully characterized, alternatives to live probiotics within antibiotic-reduction strategies for aquaculture. Progress toward commercial application will depend on resolving key questions related to dose–response relationships, processing stability in formulated feeds, and species-specific efficacy.

## 1. Introduction

Global aquaculture production reached 130.9 million tonnes in 2022, with an estimated value of USD 313 billion, and now accounts for more than 57% of fish consumed by humans [1]. This rapid growth has strengthened the role of aquaculture in global food security, but it has also increased the need for effective disease prevention, biosecurity, and nutritional health management across finfish and crustacean production systems [1]. Infectious diseases remain one of the major constraints to sustainable aquaculture development, particularly under intensive farming conditions where high stocking densities, environmental fluctuations, and handling stress can increase host susceptibility and facilitate pathogen transmission. Bacterial pathogens, including *Aeromonas hydrophila*, *Streptococcus agalactiae*, *Vibrio harveyi*, *Vibrio parahaemolyticus*, and *Piscirickettsia salmonis*, continue to cause substantial economic losses in freshwater, brackish-water, and marine aquaculture. Viral agents such as infectious spleen and kidney necrosis virus (ISKNV), nervous necrosis virus (NNV), and white spot syndrome virus (WSSV) can also cause severe episodic mortality in intensive production systems [2]. In many regions, particularly South and Southeast Asia, therapeutic antibiotics have remained an important tool for managing bacterial diseases.

Global antimicrobial use in aquaculture was estimated at approximately 10,259 tonnes in 2017 and is projected to reach around 13,600 tonnes by 2030, representing a relative increase of 33% [3]. This use is unevenly distributed, with the Asia-Pacific region accounting for more than 93.8% of total antimicrobial consumption and China alone contributing 57.9% [3]. Sustained antibiotic pressure can create favorable conditions for the selection and spread of antimicrobial resistance through chromosomal mutations and horizontal gene transfer among bacterial populations, including *Vibrio*, *Aeromonas*, and *Streptococcus* isolates from Asian aquaculture systems. Multiple antibiotic resistance indices above 0.2 are commonly reported, and multidrug resistance to tetracyclines, quinolones, and sulfonamides has become increasingly widespread [4,5]. These trends raise concerns not only for aquatic animal health and farm productivity, but also for environmental microbiomes, food-chain safety, and the long-term effectiveness of antimicrobial therapies.

Live probiotic supplementation is among the most extensively studied alternatives to antibiotics in aquaculture. The International Scientific Association for Probiotics and Prebiotics (ISAPP) definition of probiotics as live microorganisms that confer health benefits when administered in adequate amounts has provided the conceptual basis for numerous studies reporting improved growth performance, immune responses, microbiome diversity, and survival across a wide range of aquaculture species [6]. Despite these potential benefits, consistent commercial application of live probiotics remains constrained by several factors: (a) loss of viability during high-temperature pelleting and extrusion; (b) limited or transient persistence in the gastrointestinal tract; (c) strain- and host-dependent efficacy; (d) biosafety concerns, including the potential transfer of antimicrobial-resistance determinants from unsuitable strains; and (e) variable regulatory requirements among jurisdictions. For example, aquafeed pelleting and extrusion, which often involve temperatures of 80–130 °C, can substantially reduce probiotic viability and compromise reliable delivery in finished feeds [7]. Many probiotic strains also show only transient colonization in teleosts, with populations often declining within 7–14 days after supplementation because of gastrointestinal passage, bile exposure, and competition with resident microbiota [8]. In addition, the potential horizontal transfer of antimicrobial-resistance genes from probiotic strains to pathogenic or opportunistic bacteria remains an important biosafety concern, particularly in open aquaculture systems where resistant bacteria and mobile genetic elements may disseminate through water and farm effluents.

Interest in non-viable microbial preparations developed from observations that some probiotic-associated effects could persist even after microbial viability was lost, suggesting that cell-associated structures and other microbial components may retain biological activity independently of replication. This concept contributed to the introduction of the term “paraprobiotic” by Taverniti and Guglielmetti in 2011 for non-viable microbial cells or crude cell extracts that can confer health benefits [9]. The paraprobiotic concept therefore predates the 2021 ISAPP consensus definition of postbiotics. Under the current framework, some paraprobiotic preparations may also meet the definition of a postbiotic when they contain inanimate microorganisms and/or their components and confer a demonstrated health benefit on the host. Postbiotics and paraprobiotics have since attracted increasing attention as non-viable microbial-based alternatives. Postbiotics generally encompass a broader range of inactivated microbial cells, cell fragments, metabolites, and cell-free fractions, whereas paraprobiotics more specifically emphasize non-viable whole-cell preparations that retain microbial-associated molecular patterns (MAMPs) capable of interacting with host pattern-recognition receptors (PRRs) [8]. These preparations may address several practical limitations associated with live probiotics by improving feed-processing stability, compositional consistency, and biosafety. Nevertheless, although postbiotics and paraprobiotics have been increasingly studied in human nutrition and food science, their characterization, functional standardization, and mechanistic validation in aquaculture remain incomplete.

Current aquaculture studies often report fragmented endpoints, such as growth performance, immune gene expression, survival, or disease resistance, without fully integrating structural class, receptor-level mechanisms, and delivery technology. For example, limited attention has been given to how specific microbial components, including cell-wall fractions, short-chain fatty acids, extracellular vesicles, and outer membrane vesicles, interact with Toll-like receptors, NOD-like receptors, C-type lectin receptors, or other innate immune pathways in fish and crustaceans [8,10,11,12]. Similarly, few studies have systematically evaluated whether these preparations remain stable during feed manufacture, storage, and gastrointestinal transit, or whether encapsulation and other delivery strategies can improve their functional availability in commercial diets. These gaps limit the ability to compare studies, define effective products, and translate promising experimental findings into practical farm-level applications.

This review considers the fish and crustacean gut microbiome as a source of immunologically active microbial products and examines the potential role of postbiotics and paraprobiotics in antibiotic-sparing disease management in aquaculture. Rather than simply cataloguing feed additives, it critically synthesizes current evidence to clarify terminology, organize aquaculture-relevant preparations by structural class and delivery constraints, evaluate mechanistic links with PRR signaling, intestinal barrier function, immunometabolic regulation, and trained-immunity-like responses, and identify the formulation, regulatory, and experimental requirements needed for commercial translation.

## 2. Definitions, Classification, and Conceptual Framework

### 2.1. ISAPP Postbiotic Consensus and Terminological Evolution

Before the 2021 consensus statement from the ISAPP, the term “postbiotic” lacked a universally accepted definition. At least six different usages were in circulation, referring variously to microbial metabolites, fermentation by-products, heat-killed cells, and cell-free supernatants, often without a standardized requirement for either demonstrated health benefit or the presence of microbial cell material [13]. The 2021 ISAPP consensus standardized the definition of postbiotics by requiring two essential features: non-viable microbial biomass, such as whole inactivated cells, cell fragments, or cellular components, with or without metabolites; and a verified health benefit in the target host. The preparation may contain microbial metabolites together with the inanimate microorganisms or their components; however, purified metabolites or other isolated microbial products administered independently do not by themselves meet the ISAPP definition. Thus, purified short-chain fatty acids (SCFAs), bacteriocins, or other substantially purified microbial products should be considered microbial-derived compounds rather than postbiotics when administered alone [13,14]. Vinderola et al. (2024) further clarified that postbiotics differ from conventional feed additives because they require microbial biomass, distinguishing them from isolated pharmaceutical-like metabolites while allowing a range of inactivation methods [15]. The ISAPP framework does not prescribe a single inactivation method, but the final preparation must contain inanimate microorganisms and/or their components and retain the biological properties required to confer the demonstrated health benefit.

**Figure 1:**
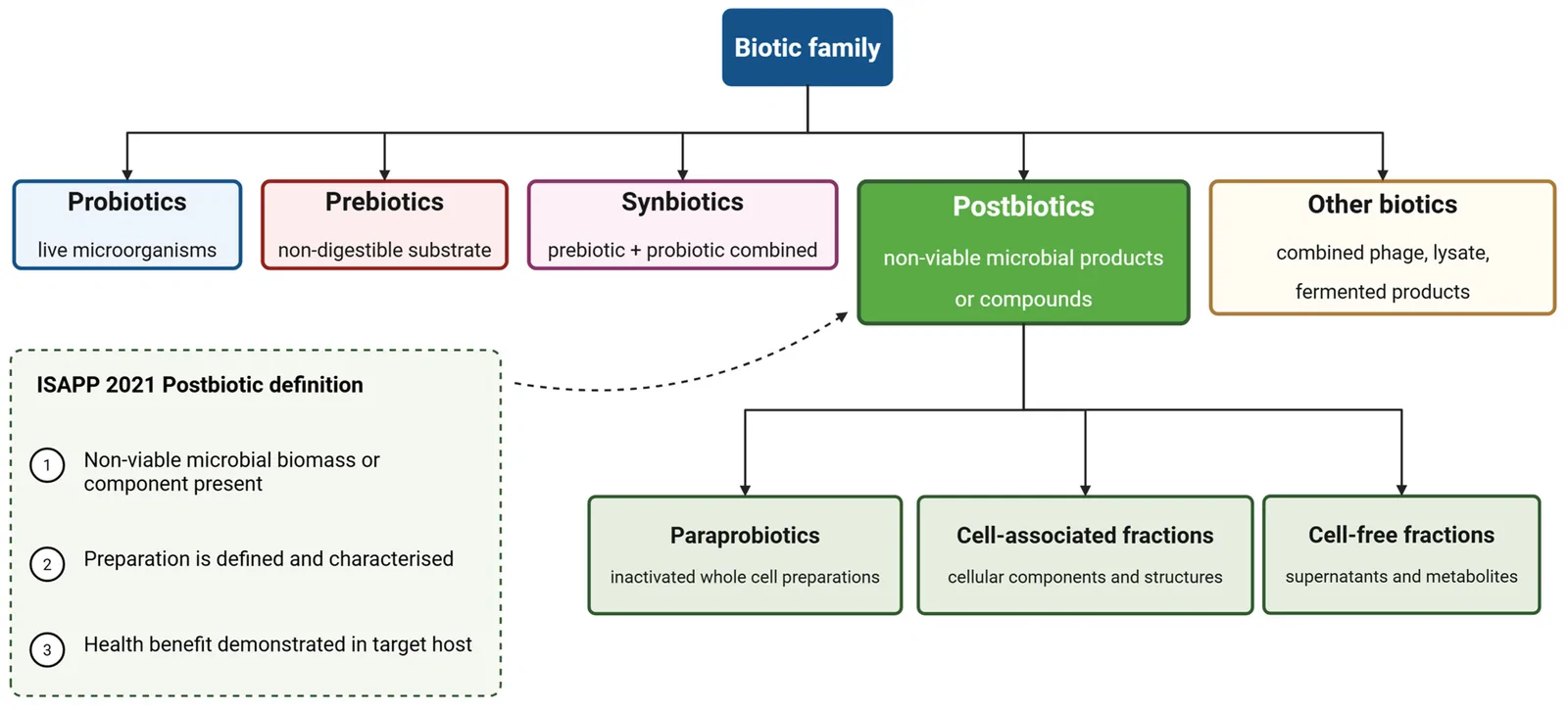
Unified classification of the biotics family and conceptual framework for postbiotic subtypes.

The term “paraprobiotic” was introduced earlier by Taverniti and Guglielmetti in 2011 to describe non-viable microbial cells or crude cell extracts that can confer health benefits [9]. The paraprobiotic concept therefore predates the standardized 2021 ISAPP postbiotic definition and should not be considered historically as a subtype of postbiotics. However, the two concepts can overlap: a paraprobiotic preparation may also meet the current ISAPP definition of a postbiotic when it contains inanimate microorganisms and/or their components and confers a demonstrated health benefit on the host. Paraprobiotics generally emphasize non-viable whole-cell or structurally preserved cellular preparations (Figure 1). These formulations retain MAMPs, including peptidoglycan (PGN), which can be recognized by NOD1/2 and Toll-like receptor (TLR), i.e., TLR2; lipoteichoic acid (LTA), which is associated with TLR2 activation; and β-glucan-type exopolysaccharides (EPS), which may interact with C-type lectin receptors, including Dectin-1 [9]. Preservation of multiple structural components may allow broader PRR engagement than more extensively disrupted or fractionated microbial preparations.

Teame et al. (2020) reviewed *Lactobacillus*-derived paraprobiotic preparations and reported TLR2-mediated NF-κB responses primarily in mammalian immune-cell models [16]. In these systems, intact inactivated cells sometimes produced stronger immunostimulatory responses than fragmented lysates, suggesting that preservation of cell-wall structure can influence biological activity. These findings are relevant to the selection of inactivation methods for paraprobiotic preparation, although direct receptor-level confirmation in aquaculture species remains more limited.

### 2.2. Structural Classification of Non-Viable Microbial Preparations and Related Products in Aquaculture

To remain consistent with the 2021 ISAPP definition, this review distinguishes qualifying postbiotic preparations from related microbial-derived products that are relevant to aquaculture. Qualifying postbiotics contain inanimate microorganisms and/or their components and confer a demonstrated health benefit, whereas SCFAs, organic acids, bacteriocins, EPS, proteins or peptides, and other substantially purified microbial products do not independently meet this definition when administered alone [13,14,15]. These compounds may nevertheless contribute to the biological activity of a postbiotic when retained within a preparation containing inanimate microbial cells or cellular components. These products should also be distinguished from prebiotics. SCFAs are microbial fermentation end-products rather than substrates selectively utilized by host microorganisms and therefore are not themselves prebiotics. In contrast, some microbial polysaccharides, including selected EPS and β-glucans, may exhibit prebiotic activity when selective utilization by host microorganisms and a resulting health benefit are demonstrated. Available reviews and experimental studies indicate that these preparations differ in their structural composition, pattern-recognition receptor (PRR) targets, processing stability, and level of supporting evidence in challenge models. For clarity, the preparations discussed in this review are grouped into broader classes based on their main structural or compositional features, including inactivated cellular preparations, cell-wall or structural components, extracellular vesicles, functional proteins, and microbial metabolites (Table 1).

### 2.3. Paraprobiotic Preparation and Classification: Inactivation Methods and MAMP Preservation

Paraprobiotic preparations can be classified partly according to the method used to eliminate microbial viability and the extent to which the treatment preserves cellular structure (Table 2). Their biological activity depends not only on complete inactivation but also on the retention and accessibility of MAMPs and other bioactive components. Relevant structures may include PGN, lipoteichoic acid (LTA), EPS, lipoproteins, and other cell-envelope-associated molecules. Therefore, the choice of processing method can influence both product safety and biological activity by altering cell integrity, MAMP exposure, and protein stability [28,29].

Thermal processing is one of the most practical approaches for deliberate microbial inactivation. Heat damages cellular membranes, nucleic acids, ribosomes, enzymes, and other essential cellular structures, resulting in loss of viability [28]. However, increasing treatment intensity can also denature heat-sensitive proteins and alter surface-associated components. Relatively heat-resistant structures, including PGN, LTA, and some polysaccharides, may be retained, although the extent of preservation depends on the strain and treatment conditions [29].

Non-thermal methods may provide greater preservation of selected cellular structures. UV irradiation primarily damages microbial nucleic acids and prevents replication, whereas ionizing radiation can produce more extensive nucleic-acid damage while maintaining substantial cell-envelope integrity under appropriate conditions [30,31]. High-pressure processing provides another non-thermal alternative but may still alter membrane permeability and cellular structure; therefore, both residual viability and structural integrity should be validated for the intended preparation [32].

Chemical inactivation can effectively eliminate microbial viability but may modify cell-surface proteins or other structural components and may raise residue concerns for routine feed application. In contrast, sonication and bead beating are primarily cell-disruption or fractionation approaches rather than dedicated whole-cell inactivation methods. These treatments can disrupt the cell envelope and release PGN, LTA, EPS, proteins, and intracellular components into soluble or particulate fractions [33,34,35,36]. They may therefore be useful for producing microbial lysates or component-rich preparations, but complete loss of viability should be independently confirmed.

**Figure 2:**
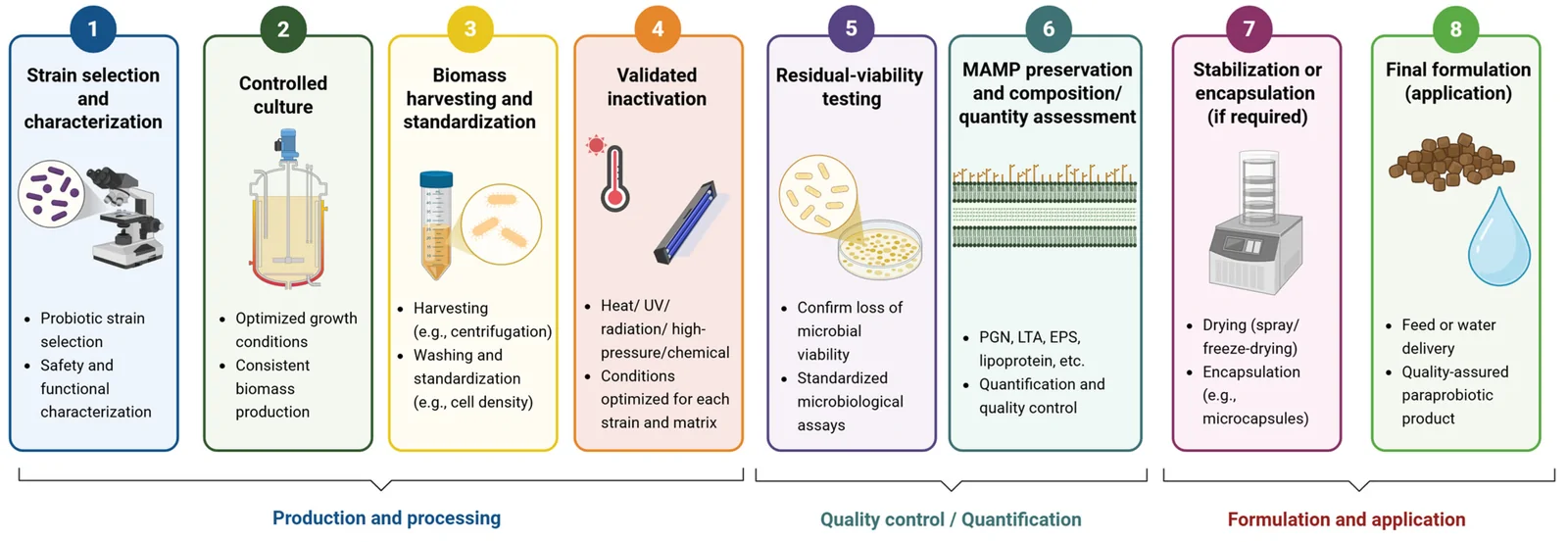
A general workflow for the production and standardization of paraprobiotic preparations.

Accordingly, selection of a processing method should balance two requirements: complete loss of microbial viability and preservation or controlled exposure of MAMPs and other bioactive components relevant to the intended function. As illustrated in Figure 2, a general production workflow includes strain selection and characterization, controlled culture, biomass harvesting and standardization, validated inactivation, residual-viability testing, assessment of MAMP preservation and product composition, quantitative characterization, stabilization or encapsulation, and final formulation. Residual viability should be confirmed after inactivation, together with assessment of product composition and relevant bioactive components. The preparation may then be stabilized or encapsulated before incorporation into feed or water-delivery formulations. Because microbial susceptibility and structural stability differ among strains and matrices, treatment conditions should be optimized and validated for each preparation rather than applied as universal parameters.

**Table 2:** Comparison of inactivation methods for paraprobiotic preparation.

Method	Primary Role	Cell-Wall/MAMP Preservation	Protein Status	Practical Considerations	Main Limitation	References
Thermal inactivation	Inactivation	Relatively heat-resistant structures such as PGN, LTA, and some EPS may be retained	Partial denaturation may occur	Simple, practical, and scalable	Excessive heat may alter surface structures and bioactive proteins	[28,29,36,37,38]
UV irradiation	Inactivation	Cell-envelope structure may be relatively preserved while nucleic acids are damaged	Generally limited thermal damage	Non-thermal and relatively simple	Penetration and effective dose depend on cell density and matrix	[30,31,38,39,40]
Ionizing radiation	Inactivation	Can retain substantial cell-envelope integrity while damaging nucleic acids	Less thermal denaturation than heat processing	Good penetration; can treat sealed preparations	Requires specialized facilities and dose validation	[30,31,41,42,43,44]
High-pressure processing	Inactivation	May preserve heat-sensitive components, although membranes can be altered	Limited heat-related denaturation	Non-thermal approach	Residual viability and structural integrity require validation	[32]
Sonication/bead beating	Disruption/fractionation	Disrupts intact cellular architecture and releases cell-wall and intracellular components	Variable	Useful for producing lysates or component-rich fractions	Complete inactivation should not be assumed; residual viability must be tested	[33,34,35,36]
Chemical inactivation	Inactivation	Surface structures may be modified or cross-linked depending on the reagent	Proteins may be altered	Effective for eliminating viability	Structural modification and residue concerns may limit routine feed use	[38,45]

PGN, peptidoglycan; LTA, lipoteichoic acid; EPS, exopolysaccharide.

## 3. Mechanisms of Action: Gut Epithelium and Immune Cell Interactions

### 3.1. Gut Architecture and Immune Surveillance in Teleosts and Crustaceans

**Figure 3:**
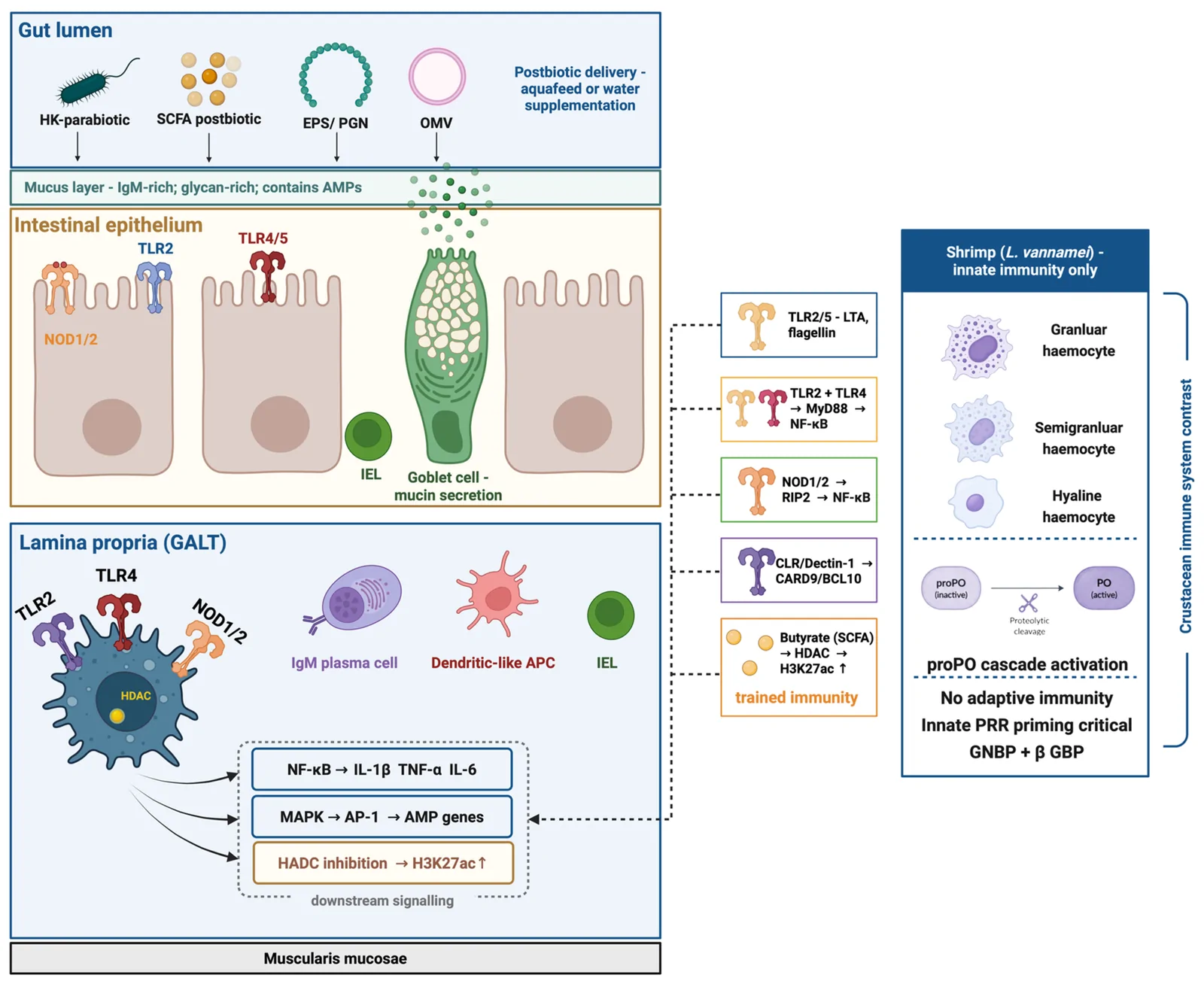
Teleost gut immune architecture and postbiotic interaction sites.

The intestine represents an important interface between microbial-derived preparations and the host immune system in both teleosts and crustaceans, although their immune organization differs substantially. In teleost fish, unlike mammals, organized Peyer’s patches are absent, but a diffuse gut-associated lymphoid tissue (GALT) is distributed throughout the intestinal lamina propria. This mucosal immune compartment includes IgM-secreting plasma cells, IgT/IgZ-secreting plasma cells, intraepithelial lymphocytes (IELs), macrophages, and dendritic cell-like antigen-presenting cells, all of which contribute to continuous sampling of luminal microbial signals and microbial-derived compounds (Figure 3) [46]. The teleost intestine contains B-cell and plasma-cell populations associated with IgM, IgD, and IgT/IgZ [47]. Among these isotypes, IgT/IgZ is particularly specialized for mucosal immunity and has functional similarities to mammalian secretory IgA, whereas IgM also contributes substantially to mucosal antibody responses. IgD is present in teleost B-cell compartments and has also been implicated in mucosal immunity, although its intestinal functions remain less well defined and may vary among species.

The teleost intestinal epithelium is composed mainly of absorptive enterocytes with well-developed microvilli, goblet cells that produce a glycan-rich mucus layer, enteroendocrine cells, and rodlet cells. Unlike the IgA-dominated mucus layer of mammals, teleost mucus contains abundant IgM as well as mucosal IgT/IgZ, reflecting the distinct organization of fish mucosal immunity. Rodlet cells, a piscine-specific cell type, have been implicated in innate defense and inflammatory responses, although their precise function remains incompletely defined [48].

In contrast, in Pacific white shrimp (*Litopenaeus vannamei*) and other penaeid shrimp, the intestinal immune system differs fundamentally from that of teleosts. Crustaceans lack immunoglobulin-based adaptive immunity, and their immune defense depends primarily on innate cellular and humoral mechanisms. Haemocytes, including granular, semigranular, and hyaline populations in the haemolymph, serve as the principal immune effector cells, while the prophenoloxidase (proPO) activation cascade represents a key innate immune amplification pathway [49]. Toll receptors and their downstream signaling components have also been characterized in several crustaceans, particularly penaeid shrimp. In contrast to teleost TLR signaling, which operates within an immune system containing both innate and adaptive components, crustacean Toll signaling contributes primarily to broad innate defense through pathways involving MyD88, NF-κB-related factors, and antimicrobial effector responses. However, ligand recognition and receptor-specific functions remain less completely resolved in crustaceans, and direct equivalence with teleost TLR pathways should therefore be interpreted cautiously [49]. Because penaeid shrimp do not possess antibody-mediated clonal memory, enhanced pathogen resistance must rely on modulation of innate immune effectors, immune priming-like responses, and improved barrier function rather than conventional adaptive immune responses [50,51]. This biological context makes the rational design of innate immune-priming postbiotic and paraprobiotic strategies particularly relevant for crustacean aquaculture.

### 3.2. Toll-Like Receptor Signaling Pathways in Teleosts

Teleost fish possess a substantially expanded TLR repertoire compared with mammals, reflecting the presence of both conserved mammalian-like TLRs and several teleost-specific forms. Up to 20 distinct TLR types have been identified in teleosts, including TLR1–5, TLR7–9, TLR13, TLR14, TLR18–23, and TLR25–28, whereas most mammals possess approximately ten canonical TLRs, TLR1–10 [52]. A systematic review by Liao and Su (2021) showed that core MyD88-dependent signaling downstream of TLR1, TLR2, TLR4, TLR5, and TLR9 is broadly conserved across teleost species and is structurally and functionally similar to the mammalian paradigm [53]. In contrast, TRIF-dependent signaling diverges more in teleosts. Mammalian TRAM, which links TLR4 to TRIF, is absent in sequenced fish genomes, so adaptor usage downstream of TLR3 and TLR4 differs from mammals [52]. Mahapatra et al. (2023) further reviewed TLR diversity across key farmed species and reported that TLR2 and TLR4 are broadly expressed in mucosal and immune-related tissues, including the intestinal epithelium, head kidney, spleen, and gills of Nile tilapia (*Oreochromis niloticus*) and Atlantic salmon (*Salmo salar*) [54]. These observations are consistent with reports that TLR2 and TLR4 are constitutively expressed in multiple internal organs and barrier tissues, supporting their roles in sensing microbial components during aquaculture-relevant infections.

Among the MAMPs, Gram-positive bacterial cell-wall components are particularly relevant to TLR2-mediated recognition in fish, where TLR2 is an important pattern-recognition receptor involved in innate immune signaling. These molecules are important immunostimulatory components of *Bacillus*-derived paraprobiotics and postbiotics, which deliver inactivated cell-wall structures or secreted microbial products rather than live bacteria [55]. Xu et al. (2025) [56] demonstrated that dietary supplementation with PGN and LTA extracted from *Bacillus pumilus* SE5 activated the intestinal TLR2/MyD88 signaling axis in grouper (*Epinephelus coioides*). This activation was associated with significant upregulation of pro-inflammatory and immune-related markers, including interleukin-1β (IL-1β), interleukin-8 (IL-8), and lysozyme, consistent with engagement of the canonical TLR2–MyD88–NF-κB pathway. Basu et al. (2012) also demonstrated constitutive ontogenic expression of TLR2 and strong inducible responses to lipopeptide stimulation in Indian major carp mrigal (*Cirrhinus mrigala*), supporting the conservation of this pathway in cyprinid teleosts [57]. These findings implicate the TLR2–MyD88–NF-κB axis as an important proximal signaling route through which *Bacillus*-origin paraprobiotics may exert immunostimulatory and resilience-enhancing effects in teleosts, particularly at the intestinal interface.

In teleosts, lipopolysaccharide (LPS) recognition through TLR4 is more complex and appears to be species-dependent. Rebl et al. (2010) reported that TLR4 orthologues in bony fish have diverged substantially in ligand specificity, with some species showing evidence of LPS/MD-2-like recognition, whereas others display non-canonical or regulatory functions [58]. In zebrafish (*Danio rerio*), LPS responses occur largely through TLR4- and MyD88-independent mechanisms. The two zebrafish TLR4 paralogues have been shown to suppress, rather than activate, MyD88-dependent NF-κB signaling, suggesting a regulatory rather than classical pro-inflammatory receptor function [59]. By contrast, grass carp (*Ctenopharyngodon idella*) and common carp (*Cyprinus carpio*) possess TLR4 variants that can mediate responses to LPS and to heat-shock proteins released from necrotic cells, consistent with roles in both infection-associated and sterile inflammation in economically important cyprinid species [60]. These species-specific differences indicate that TLR4-mediated responses to Gram-negative bacterial products should not be generalized across teleosts without experimental validation in the target host.

TLR9 is an endosomal DNA-sensing receptor that recognizes unmethylated CpG dinucleotides, which are abundant in bacterial genomes but relatively uncommon in vertebrate host DNA. In paraprobiotic and postbiotic preparations, bacterial DNA may become available when probiotic cells are partially lysed during inactivation, such as by heat, pressure, or irradiation, or during gastrointestinal digestion. Once internalized into endosomal compartments, CpG-rich bacterial DNA can engage TLR9 and contribute to downstream innate immune activation [61]. This pathway is particularly relevant for non-viable microbial preparations because inactivation and digestion may expose intracellular ligands that are less accessible in intact viable cells.

### 3.3. NOD-Like Receptors and Peptidoglycan Postbiotic Ligands

NOD1 and NOD2 function as cytoplasmic sensors of peptidoglycan-derived muropeptides, which may enter fish enterocytes through apical uptake mechanisms, endocytosis, or epithelial sampling pathways. NOD2, in particular, is highly conserved in teleosts and recognizes muramyl dipeptide (MDP), the minimal immunologically active peptidoglycan motif shared by both Gram-positive and Gram-negative bacteria. This pathway has been well characterized in model and aquaculture-relevant species, including zebrafish and Nile tilapia [52]. Following MDP recognition, NOD2 oligomerizes and recruits receptor-interacting protein kinase 2 (RIP2), leading to activation of NF-κB and MAPK signaling pathways. These cascades promote the transcription of pro-inflammatory and immune-regulatory mediators, including IL-1β, TNF-like cytokines, and AP-1-dependent genes involved in host defense, epithelial repair, and inflammatory regulation [53].

Importantly, low to moderate doses of MDP may induce a primed innate immune phenotype in fish macrophages without causing overt inflammatory pathology. This response is consistent with a trained-immunity-like state, in which innate immune cells show enhanced responsiveness to secondary stimulation after prior exposure to microbial ligands. Petit et al. (2019) demonstrated in carp macrophages that sub-inflammatory exposure to MDP or whole-bacterial peptidoglycan enhanced responses to later stimulation through metabolic and epigenetic reprogramming [62]. In this context, MDP and related peptidoglycan fragments present in PGN-rich postbiotic fractions from *Bacillus* or *Lactobacillus* preparations may contribute to controlled innate immune priming in fish. At appropriate doses, these ligands could enhance pathogen resilience while limiting excessive inflammation, although dose–response relationships and species-specific effects require further validation in aquaculture models.

### 3.4. C-Type Lectin Receptors and β-Glucan Recognition in Teleosts

β-glucans derived from fungal or yeast cell walls represent one of the most extensively characterized classes of postbiotic ligands in aquaculture. These molecules act primarily through C-type lectin-like receptors (CLRs) and complement receptor 3 (CR3)-associated pathways in teleosts. Petit et al. (2019) [18] demonstrated that β-glucan stimulation of *Cyprinus carpio* macrophages upregulated genes involved in the CLR–CARD9/BCL10 signaling pathway, together with NF-κB and MAPK-associated genes, respiratory burst-related genes, and pro-inflammatory cytokines. These findings support the functional conservation of this CLR-associated signaling module in teleost innate immunity.

CR3 has also been identified in fish phagocytes as a co-receptor involved in β-glucan recognition, particularly during the detection of β-glucan-coated or opsonized particles. Machuca et al. (2022) reviewed the immunomodulatory effects of yeast β-glucans in teleost species, including salmonids, tilapia, and marine fish, and reported consistent activation of innate immune markers such as oxidative burst, lysozyme activity, and complement C3 expression [17]. These responses suggest that β-glucan-mediated CLR and CR3 pathways can strengthen broad innate immune defenses against bacterial and viral challenges in aquaculture systems.

Beyond direct receptor-mediated immune activation, β-glucans may also influence host immunity indirectly through effects on the intestinal microbiota and microbial metabolite production. Studies in common carp and other teleost models indicate that dietary β-glucan can modulate gut microbial composition and increase SCFA production, including acetate, propionate, and butyrate [18,52]. These metabolites may contribute to intestinal barrier regulation, immunometabolic signaling, and epigenetic modulation of innate immune responses. Thus, β-glucan activity in fish likely involves both direct CLR/CR3-mediated recognition by immune cells and indirect microbiota-dependent effects. However, the relative contribution of these pathways remains incompletely defined and should be investigated using integrated studies that combine receptor-level assays, microbiome profiling, metabolomics, and pathogen challenge models.

### 3.5. SCFA, Epigenetic Regulation, and the Trained Immunity Hypothesis

**Figure 4:**
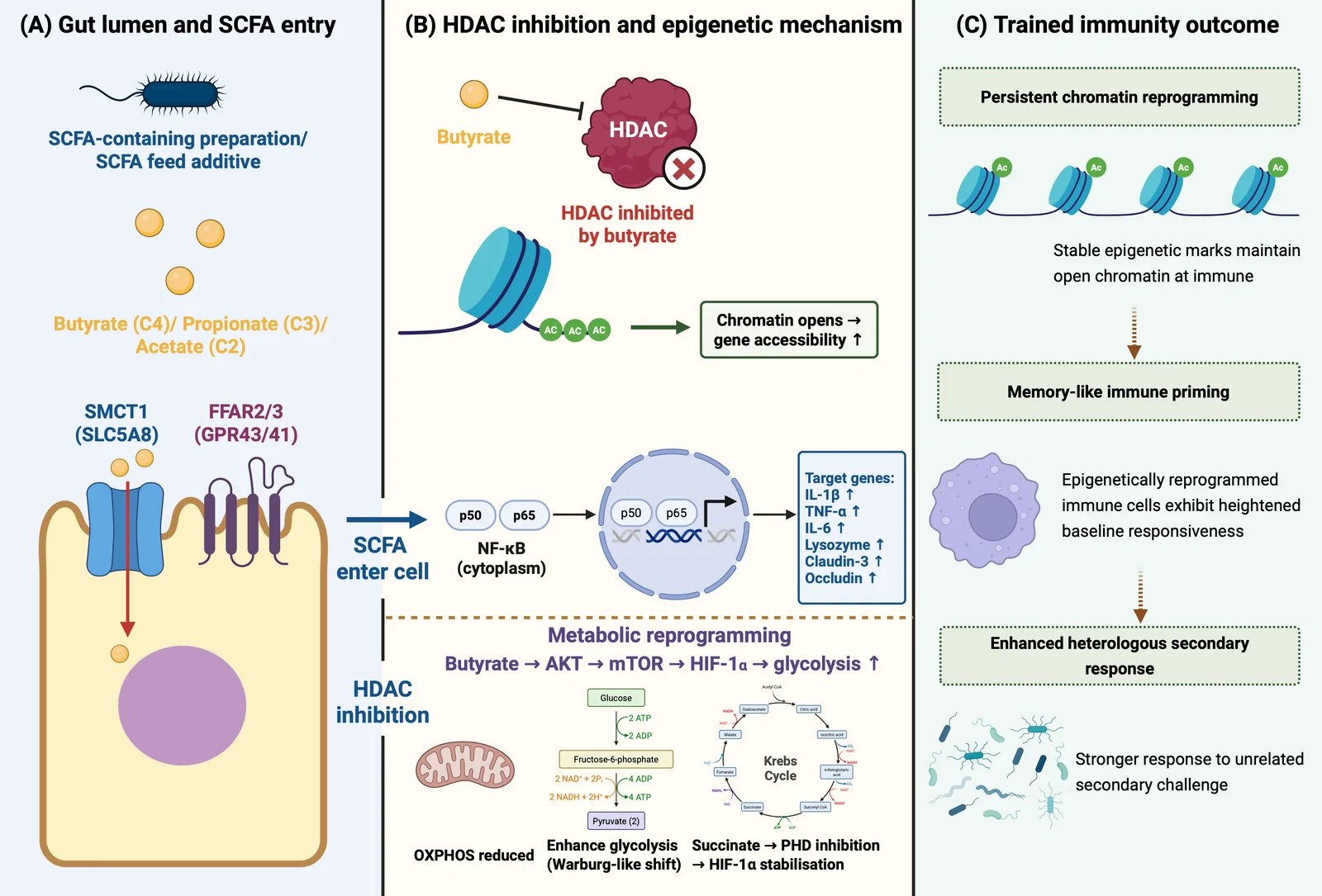
Proposed mechanistic framework of SCFA-mediated trained immune priming in teleost intestinal immune cells, showing (**A**) SCFA production and cellular entry, (**B**) histone deacetylase (HDAC) inhibition with epigenetic and metabolic reprogramming, and (**C**) trained-immunity-related outcomes. The mechanisms shown in panels (**A**,**B**) are supported by experimental evidence in fish, whereas the persistent trained-immunity response illustrated in panel (**C**) remains proposed and has not yet been directly validated in teleost immune cells.

SCFAs, particularly butyrate, are physiologically relevant microbial metabolites in the intestinal lumen and can function as histone deacetylase (HDAC) inhibitors (Figure 4). Butyrate preferentially inhibits class I and class II HDACs, preventing the removal of acetyl groups from histone lysine residues and thereby promoting histone hyperacetylation. In immune cells, butyrate-induced histone acetylation is often associated with active chromatin marks, including H3K27ac and H3K9ac, which are enriched at promoters and enhancers of immune-related genes. These changes can increase chromatin accessibility, facilitate transcription factor binding, and enhance the transcriptional responsiveness of cytokine and host-defense genes [63,64].

In mammalian monocytes and macrophages, HDAC inhibition by butyrate is considered one mechanism contributing to the epigenetic reprogramming that underlies trained immunity. Trained immunity, as defined by Netea et al. (2016, 2020), refers to a functional state in which innate immune cells previously exposed to an initial stimulus, such as β-glucan, BCG vaccination, or muramyl dipeptide, exhibit enhanced responsiveness to later heterologous challenges through persistent epigenetic modifications and metabolic reprogramming [63,64]. Whether an analogous SCFA-driven epigenetic priming mechanism operates in teleost innate immune cells remains to be directly demonstrated. Current evidence in fish is suggestive but largely indirect, relying mainly on changes in histone-modifying gene expression and immune-related transcription rather than chromatin-level assays in pathogen-challenged immune cells.

In aquaculture models, stronger evidence for trained-immunity-like responses has been generated using β-glucan and muramyl dipeptide. Petit et al. (2019) demonstrated that primary exposure of common carp head kidney-derived macrophages to β-glucan or MDP induced a trained phenotype that persisted after removal of the initial stimulus. These trained macrophages showed enhanced phagocytosis, increased expression of IL-6 and TNF-α, elevated reactive oxygen and nitrogen species production, and a metabolic shift from oxidative phosphorylation toward glycolysis, features that closely parallel mammalian trained immunity [62]. Waikhom et al. (2022) further supported this concept by confirming β-glucan-induced trained-immunity markers in carp, providing additional evidence that this mechanism may be conserved in cyprinid teleosts [65].

Dietary butyrate and propionate may contribute to related immune-priming effects by modulating HDAC activity in fish intestinal epithelial cells and associated immune cells. Through this mechanism, SCFAs could promote persistent histone acetylation patterns that increase the responsiveness of immune and barrier-related genes, although this remains a hypothesis that requires direct validation in fish. In European sea bass and other aquatic species, sodium butyrate administered as a feed additive has been shown to influence histone acetylation patterns and regulate the expression of epigenetic regulatory genes, including *hdac11*, *ehmt2*, and *dicer1*. These changes have been associated with improved mucosal protection and immune homeostasis [66,67]. Tran et al. (2023) also reported that butyrate and *Clostridium butyricum* exert broad effects across aquatic animals, including upregulation of tight-junction genes such as *claudin*, *occludin*, and *ZO-1*, increased antimicrobial peptide expression, and modulation of pro-inflammatory and regulatory cytokine genes [22]. These transcriptional changes are consistent with strengthened intestinal barrier function, improved pathogen exclusion, and enhanced disease resistance. However, under the strict ISAPP framework, purified SCFAs alone should be regarded as microbial metabolites rather than postbiotics unless they are present within a preparation containing non-viable microbial biomass. Therefore, SCFA-containing postbiotic preparations and SCFA-based feed additives should be distinguished conceptually, even though both may contribute to immunomodulatory and barrier-supporting effects in aquaculture [67,68,69].

### 3.6. Gut Barrier Function Enhancement by Postbiotics

In addition to direct pattern-recognition receptor signaling, several postbiotic-related preparations can improve intestinal barrier integrity in fish through complementary molecular and cellular mechanisms. SCFAs, particularly butyrate, have been shown to regulate tight-junction protein genes, including *claudin-3*, *occludin*, and *ZO-1*, in the teleost intestinal epithelium. By strengthening tight-junction organization, these metabolites may reduce paracellular permeability and limit bacterial translocation into the lamina propria [22,70]. Abdel-Tawwab et al. (2021) demonstrated that dietary sodium butyrate nanoparticles at 1.0–1.5 mg/kg feed improved intestinal morphology in Nile tilapia, including increased villus height-to-crypt depth ratio, which suggests enhanced absorptive surface area and nutrient uptake capacity [71]. The same study also reported upregulation of tight-junction-related genes, increased lysozyme and complement activities, and improved survival following *A. hydrophila* challenge. Similarly, Fontinha et al. (2024) [23] examined the effects of acetate, propionate, and butyrate on immune and oxidative responses in European seabass using ex vivo gut sac preparations. Their study showed that these SCFAs modulated intestinal responses to *Vibrio anguillarum* challenge, supporting their potential role as functional feed additives in marine teleost nutrition.

Other postbiotic categories, including EPS-type preparations and cell-wall-derived fractions, may further support barrier function by stimulating goblet cell activity and mucin gene expression, such as *muc2* and *muc5b*. These effects can enhance the mucus layer and strengthen the first physical barrier between luminal microorganisms and the intestinal epithelium. In parallel, β-glucan- and lipoteichoic acid-containing preparations have been associated with increased expression of antimicrobial peptides, including piscidin, β-defensin, and NK-lysin, in teleost mucosal tissues [17,54]. Such responses may contribute to colonization resistance by limiting the attachment, growth, or invasion of bacterial pathogens at the epithelial surface.

This multilayered barrier reinforcement, involving tighter epithelial junctions, enhanced mucus production, and antimicrobial peptide induction, represents one of the more practically relevant benefits of postbiotic-based approaches in aquaculture. In intensive production systems, fish are frequently exposed to stressors such as high stocking density, poor water quality, handling, pathogen pressure, and anti-nutritional factors from high-plant-protein diets. These stressors can compromise epithelial integrity and increase the risk of intestinal inflammation, microbial imbalance, and systemic pathogen entry [7]. By supporting gut barrier function without relying primarily on strong inflammatory activation, postbiotic and SCFA-containing preparations may provide a useful strategy to improve intestinal resilience, survival, and productivity under high-density aquaculture conditions. However, further work is needed to define optimal doses, formulation stability, and species-specific responses before these effects can be reliably translated into commercial feeding programs [72].

## 4. Evidence from Key Aquaculture Species

### 4.1. Nile Tilapia

Nile tilapia is one of the most intensively farmed tropical finfish worldwide, with annual production exceeding 6–7 million tonnes, making it a major species in freshwater aquaculture [1]. Under intensive, high-density production conditions, Nile tilapia is particularly vulnerable to bacterial diseases, most notably to *S. agalactiae*-induced streptococcosis [73] and *A. hydrophila*-induced motile *Aeromonas* septicaemia [74]. Among aquaculture species, tilapia has one of the most developed evidence bases for SCFA-based and postbiotic-related interventions [75,76]. 

Dietary sodium butyrate supplementation, commonly tested at 0.5–2.0 g/kg feed, has been associated with improved intestinal morphology, including increased villus height and width, which may expand absorptive surface area and improve nutrient utilization. These structural changes are often accompanied by upregulation of tight-junction protein genes and increased lysozyme activity [77]. Sodium butyrate may also modulate gut microbial composition by favoring beneficial genera such as *Lactobacillus* and *Bacillus*, while reducing the relative abundance of potential pathogens [22,70]. Together, these effects can support intestinal barrier function, mucosal immunity, and disease resistance, as reflected by improved survival in bacterial challenge trials, including those using *A. hydrophila*. Abdel Rahman et al. Abdel-Tawwab et al. (2021) further showed that nanoencapsulated sodium butyrate at 1.0–1.5 mg/kg feed improved hematological immune indices, hepatic antioxidant capacity, and immune-related gene expression, including *IL-1β* and lysozyme, in Nile tilapia [71].

At the paraprobiotic level, heat-killed bacterial preparations have also shown promise. Heat-killed *Lactiplantibacillus plantarum* L-137 (formerly *Lactobacillus plantarum*) has been evaluated in tilapia and related fish models, with evidence that it can retain immunostimulatory activity and modulate cytokine responses, including *IL-1β* and *IL-10*, through pathways involving TLR2-mediated innate immune activation [78]. However, because some supporting mechanistic evidence comes from in vitro fish cell-line models or from feeding trials in non-tilapia species, its direct relevance to Nile tilapia should be interpreted cautiously. More tilapia-specific validation is needed to confirm whether these immune pathways operate consistently under commercial feeding and disease-challenge conditions.

Heat-killed *Bacillus subtilis* preparations have also been tested in Nile tilapia, often at concentrations of approximately 10^9^–10^10^ cell equivalents/g feed. These preparations have been associated with increased expression of non-specific immune markers, including *TLR2*, *MyD88*, *IL-1β*, *IL-10*, and lysozyme [22]. More recently, Linh et al. (2026) reported that dietary supplementation with encapsulated *Lysinibacillus* sp. PWR01 improved specific growth rate, antioxidant enzyme activity, immune gene expression, including *TLR2*, *MyD88*, and *IL-1β*, and post-challenge survival in Nile tilapia experimentally infected with *A. hydrophila* [79]. Genomic characterization of this strain, as well as *Bacillus* sp. KNSH11 from the same research group [80], identified bacteriocin biosynthetic clusters and CRISPR-Cas defense systems, while no known virulence factors were detected [79]. These findings provide an initial safety and functional basis for developing more defined postbiotic or paraprobiotic preparations from these candidate strains.

Despite these advances, an important gap remains in distinguishing the effects of viable cells, inactivated whole cells, and secreted microbial products from the same strain. A direct “three-arm” comparison involving the live probiotic, heat-killed paraprobiotic, and characterized cell-free supernatant or metabolite-rich fraction within the same tilapia challenge model would be especially valuable. Such a design would help clarify whether protection is primarily driven by colonization and metabolic activity, preserved structural MAMPs, or secreted bioactive compounds. To date, few studies have applied this framework rigorously in tilapia. Addressing this gap would provide a critical bridge between the current probiotic-centered approach and the development of more stable, defined, and non-viable microbial-based interventions for intensive tilapia farming.

### 4.2. Atlantic Salmon (Salmo salar) and Rainbow Trout (Oncorhynchus mykiss)

Among characterized postbiotic-related preparations evaluated in salmonid aquaculture, *Saccharomyces cerevisiae*-derived β-1,3/1,6-glucan products, particularly MacroGard, currently represent one of the strongest evidence bases for consistent innate immune enhancement in controlled trials [19,81]. MacroGard acts through conserved pattern-recognition receptors on teleost phagocytes, activating signaling pathways that resemble the Dectin-1/CARD9/BCL10 cascade described in mammals [17]. This receptor-mediated interaction has been associated with increased respiratory burst activity, enhanced phagocytic capacity in macrophages and neutrophils, elevated lysozyme activity, and upregulation of complement C3 expression, collectively strengthening the host’s ability to recognize and control invading bacterial pathogens [82,83]. These findings support the use of MacroGard as a benchmark β-glucan-based immunomodulatory preparation in salmonid studies.

Petit et al. (2016) further extended earlier observations in carp by showing that MacroGard may act not only through direct engagement of pattern-recognition receptors, particularly CLR-associated CARD9/BCL10 pathways, but also through indirect modulation of the gut microbiota [81]. This suggests that β-glucan preparations may influence salmonid immunity through both host receptor-mediated signaling and microbiota-dependent metabolic or immunological effects. However, the relative contribution of these direct and indirect pathways remains incompletely defined and requires further validation in salmonid-specific models.

In rainbow trout, Villumsen et al. (2020) highlighted the potential value of synbiotic supplementation, combining probiotic and prebiotic components, as a strategy to improve gut health, immune function, and disease resistance [84]. In triploid rainbow trout, a 0.2% inclusion rate appeared to provide a functional threshold that balanced gut microbiota modulation with systemic immune priming. The reduced mortality observed following *Yersinia ruckeri* serotype O1, biotype 2 challenge supports the prophylactic potential of this approach in salmonids [84]. Mechanistically, the study proposed that synbiotic effects on gut microbiota composition and mucosal immune indices, including lysozyme activity and localized cytokine modulation, may be complementary, with the prebiotic component supporting probiotic persistence while live cells directly interact with mucosal immune receptors [85]. Nevertheless, because synbiotics include viable microorganisms, these findings should be distinguished from evidence for non-viable postbiotic or paraprobiotic preparations. Whether the combined effects of synbiotics exceed those achievable by optimized single-component supplementation remains insufficiently resolved.

Rocha et al. (2023) evaluated heat-killed *L. plantarum* HK L-137 as a paraprobiotic in Atlantic salmon smolts and provided an important methodological reference for salmonid studies [78]. The study showed that key structural and immunostimulatory properties of HK L-137 were retained after exposure to 80 °C, a temperature relevant to feed pelleting. This finding is important because it addresses a major limitation of live probiotics: the loss of viability during feed processing and storage. By maintaining immunostimulatory activity without requiring live-cell delivery, paraprobiotic preparations such as HK L-137 may offer a more stable and biosafe alternative for salmonid aquaculture. However, claims regarding innate immune “training” should be interpreted cautiously unless supported by longitudinal functional assays, metabolic profiling, or chromatin-level evidence. Future studies should directly compare live probiotics, heat-killed whole cells, purified cell-wall fractions, and cell-free metabolites from the same strain to clarify the mechanisms underlying protection in Atlantic salmon and rainbow trout [78].

### 4.3. Pacific White Shrimp

*Litopenaeus vannamei* is currently the world’s most valuable single aquaculture species, with annual global production exceeding 5.8 million tonnes and a farm-gate value of more than USD 30 billion. Unlike teleost fish, penaeid shrimp lack lymphoid-based adaptive immunity and therefore depend on innate immune mechanisms, including haemocytes, pattern-recognition receptors (PRRs), the prophenoloxidase cascade, antimicrobial peptides, and reactive oxygen species, for pathogen defense [86,87,88]. This biological context makes shrimp particularly responsive to nutritional and environmental immunomodulation, especially under intensive production conditions where disease pressure is high.

In a 56-day feeding trial, Ballantyne et al. (2023) supplemented *L. vannamei* diets with a heat-killed postbiotic preparation of *Pediococcus pentosaceus* PP4012 at 10^5^ and 10^6^ cell equivalents/g feed [89]. The study reported significant improvements in final body weight, specific growth rate, and feed conversion ratio, together with increased gut microbial diversity based on 16S rRNA amplicon sequencing and higher total haemocyte counts compared with the control group. Shrimp receiving the higher inclusion level also showed markedly reduced cumulative mortality following challenge with an AHPND-associated strain of *V. parahaemolyticus*. These findings suggest that heat-killed lactic acid bacterial preparations can support both growth performance and innate immune readiness in shrimp.

Ren et al. (2022) evaluated dietary heat-killed *L. plantarum* at 0.05% and 0.1% inclusion levels in juvenile *L. vannamei* over 28 days [90]. Supplementation improved body weight gain, antioxidant enzyme activities, including superoxide dismutase and catalase, and intestinal morphology, as reflected by higher villus density and improved microvilli integrity. The treatment also produced favorable shifts in the gut microbiome, including enrichment of Verrucomicrobia and Firmicutes. These results indicate that inactivated *L. plantarum* may enhance shrimp performance through combined effects on oxidative balance, intestinal structure, and microbial community composition.

Wu et al. (2024) [91] provided one of the most direct comparisons between live and non-viable bacterial preparations in *L. vannamei*. Inactivated *L. plantarum* Ep-M17 was administered at doses equivalent to the live preparation and produced comparable effects on growth, gut microbiome modulation, and expression of key immune-related genes, including *proPO*, lipopolysaccharide-binding protein, and Toll-pathway genes. This direct comparison suggests that, for this strain, immunostimulatory activity is not dependent on bacterial viability and may instead be mediated by preserved structural components or non-viable microbial products.

β-glucan-enriched yeast cell-wall preparations currently have the broadest commercial adoption among postbiotic-related categories in shrimp aquaculture. These products are routinely incorporated into intensive *L. vannamei* diets in major shrimp-producing countries, including Thailand, Vietnam, Ecuador, and Indonesia [18,22]. Their widespread use, together with a relatively consistent body of controlled trial evidence, positions yeast cell-wall and β-glucan-based preparations as one of the most translationally mature non-viable microbial-based interventions for shrimp health management. Nevertheless, further work is needed to distinguish the relative contributions of β-glucans, mannans, nucleotides, and other cell-wall-associated components, as well as to define optimal inclusion levels under different culture conditions and pathogen-challenge models.

### 4.4. Asian Seabass (Lates calcarifer) and Other Marine Teleosts

Asian seabass (*Lates calcarifer*) is one of the most economically important marine aquaculture species in Southeast Asia, but its production remains constrained by several major pathogens, particularly during larval and juvenile stages. Important disease agents include NNV, *V. harveyi*, and *S. iniae* [92]. Compared with tilapia, salmonids, and shrimp, the evidence base for postbiotic and paraprobiotic applications in Asian seabass remains relatively limited. Therefore, selected findings from other marine teleosts are also considered where they provide relevant information on gut health, immune modulation, and feed-based delivery (Table 3). Several of these findings also provide useful mechanistic context for future development of postbiotic and paraprobiotic strategies in marine teleosts. For example, CpG-containing bacterial DNA released from heat-killed *Bacillus* or *Lactobacillus* preparations may provide a TLR9-dependent route for antiviral immune priming through induction of type I interferon-related responses [12,54]. This mechanism is particularly relevant for viral diseases such as NNV, although direct validation in Asian seabass challenge models is still needed.

Wisetkaeo et al. (2026) characterized caffeic acid as an immunostimulant in Asian seabass and reported improved growth performance, enhanced mucosal and systemic immune parameters, and increased resistance to *S. agalactiae* infection [99]. The study also showed upregulation of immune- and antioxidant-related genes and reduced lesion severity after challenge. Although caffeic acid is not a postbiotic, these findings provide useful evidence that innate immune pathways in Asian seabass can be nutritionally modulated. The reported involvement of NF-κB-, IRF-, and IFN-related signaling pathways suggests that PRR-associated immune priming may be a feasible strategy in this species. This provides a conceptual basis for future studies examining whether defined postbiotic or paraprobiotic preparations can induce similar protective responses.

NNV remains a major concern in Asian seabass culture, and Satyanarayana et al. (2021) highlighted the importance of sensitive diagnostic approaches for detecting this virus [100]. While diagnostics are essential for early detection and disease surveillance, preventive immunomodulatory strategies could complement these tools by strengthening early innate antiviral responses and potentially reducing viral replication or disease severity. However, the extent to which postbiotic-based interventions can reduce NNV burden or improve survival in Asian seabass has not yet been clearly established.

Evidence from other marine teleosts suggests that SCFA-based feed additives, particularly butyrate, may help mitigate intestinal dysfunction associated with intensive feeding practices. In gilthead sea bream (*Sparus aurata*), extreme plant-based diets with low fishmeal and fish oil inclusion can induce enteritis-like changes, including villus flattening, altered tight-junction organization, reduced transepithelial resistance, and inflammatory shifts in mucosal gene expression [101]. Dietary sodium butyrate at 0.4% of the diet partially or fully reversed several of these alterations by improving villus morphology, restoring expression of mucosal immune and mucus-related genes, and helping normalize distal-gut microbiome composition. These findings indicate that butyrate may support intestinal barrier function and mucosal homeostasis under high plant-protein feeding conditions.

Taken together, evidence from Asian seabass and related marine teleosts suggests that postbiotic, paraprobiotic, and SCFA-based strategies may have value for improving mucosal immunity, antiviral readiness, and gut barrier resilience. However, the current evidence remains uneven, with stronger support from other marine teleost models than from Asian seabass itself. Future studies should directly evaluate defined non-viable microbial preparations in Asian seabass using controlled feeding trials, receptor-level immune assays, microbiome and metabolite profiling, and pathogen challenge models involving NNV, *V. harveyi*, or *S. iniae*. Such work will be necessary before findings from gilthead sea bream, salmonids, or other marine teleosts can be confidently translated to Asian seabass production systems [102].

## 5. Encapsulation Technologies and Aquafeed Delivery

### 5.1. The Aquafeed Processing Challenge

Commercial aquafeed manufacturing imposes stringent stability requirements on bioactive postbiotic and paraprobiotic preparations [103]. During pelleting and extrusion, ingredients may be exposed to temperatures of approximately 80–120 °C and pressures of 15–40 bar for 20–90 s. These conditions can reduce or eliminate the viability of live probiotic cells, promote the loss of volatile compounds such as SCFAs, and partially denature protein-based bioactives [104]. Although some postbiotic effectors, including bacteriocins and heat-shock proteins, may be more thermostable than viable cells, they can still undergo partial denaturation or conformational changes at 90–120 °C, especially during prolonged exposure or under high-shear processing conditions.

Several studies on postbiotic products intended for fish feeds have shown that biological activity can be retained, or at least partially preserved, after exposure to temperatures of approximately 95–105 °C. However, activity often declines when processing temperatures exceed 130 °C or when exposure is prolonged [105]. These thermal stability profiles indicate that commercial postbiotic preparations may require formulation-level protection, including encapsulation, matrix binding, or adjustment of processing sequence, to maintain activity during fish pellet production.

**Figure 5:**
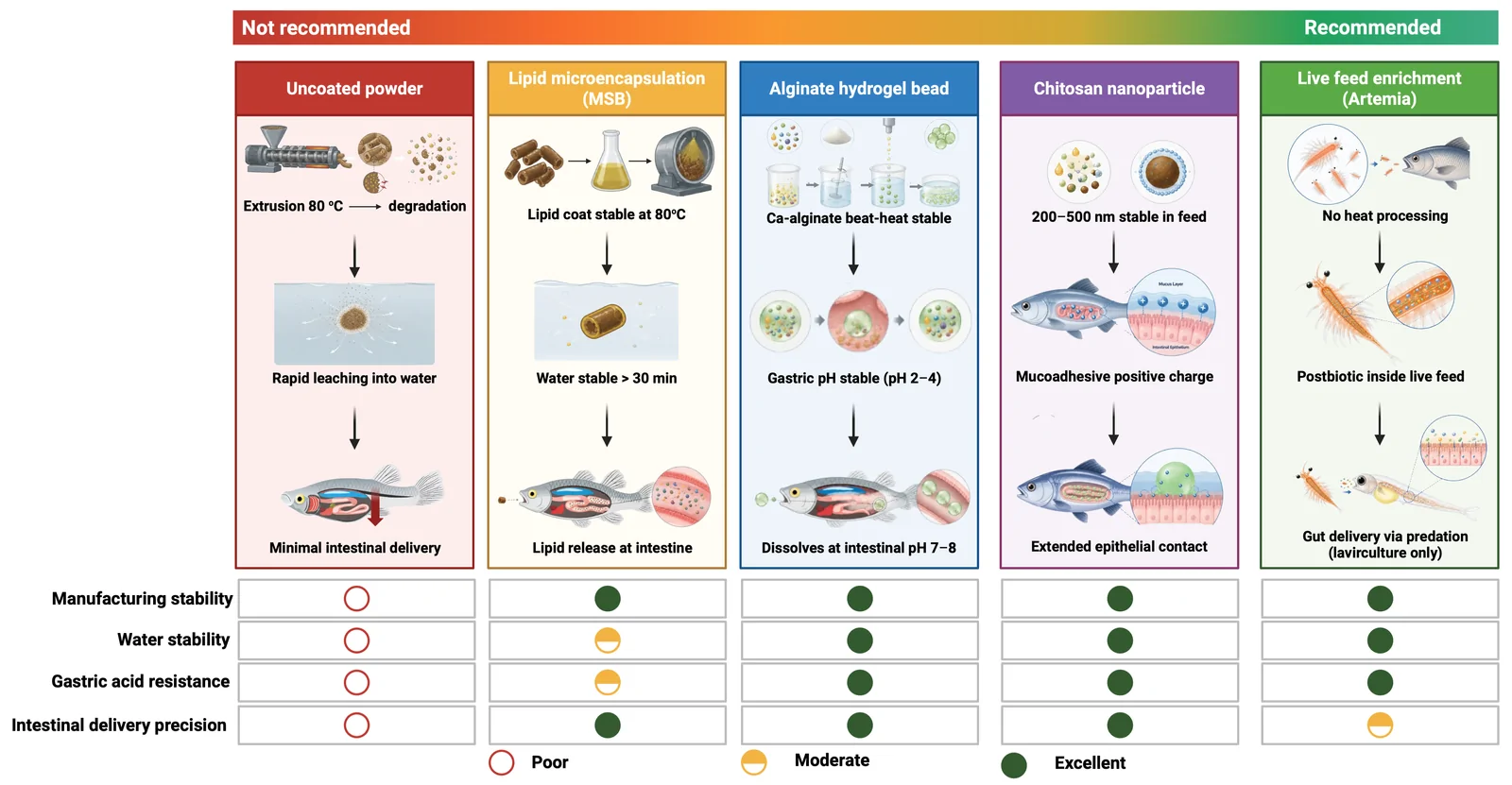
Aquafeed-based postbiotic delivery systems: comparison of encapsulation strategies and performance outcomes.

Aquatic delivery presents an additional challenge because pelleted feeds often remain in the water for 30–180 min before ingestion. During this period, unprotected water-soluble bioactives, such as SCFAs, organic acids, and exopolysaccharides, may leach into the water column before reaching the intended intestinal site of action. This loss reduces delivery efficiency and may increase variability in biological responses. Therefore, encapsulation represents a key technical requirement for many postbiotic and paraprobiotic categories in pelleted aquafeeds, particularly when the active components are water-soluble, volatile, or sensitive to heat and shear stress (Figure 5).

### 5.2. Lipid Microencapsulation for SCFA-Based Preparations

Microencapsulated sodium butyrate (MSB), typically formulated within a hydrogenated vegetable fat matrix such as palm stearin, represents one of the most commercially mature encapsulation approaches for SCFA-based feed additives in aquaculture. Similar protected butyrate formulations are already well established in terrestrial livestock nutrition [106]. Additionally, protected butyrate forms have been reviewed specifically for aquafeed use [107]. The hydrogenated-fat coating remains solid and poorly water-soluble under ambient conditions and during standard feed-processing temperatures, thereby helping to protect sodium butyrate from volatilization, premature release, and early leaching during extrusion, pelleting, and water exposure. Once ingested, pancreatic lipases and bile salts in the fish intestine promote degradation of the lipid matrix, allowing more localized release of butyrate at the mucosal surface. This controlled release can improve intestinal availability compared with uncoated sodium butyrate, which may disperse more rapidly within the pellet or leach before ingestion [108].

Controlled trials in salmonids and other farmed fish indicate that MSB can outperform equivalent doses of uncoated sodium butyrate in pelleted feed formulations, particularly when diets are exposed to high processing temperatures or prolonged water contact [109]. In such conditions, unencapsulated sodium butyrate may show substantial reductions in retained activity, whereas lipid-coated formulations provide greater protection against heat, moisture, and leaching losses. These properties make MSB especially relevant for aquafeeds used in intensive systems, where processing stability and predictable intestinal delivery are essential.

MSB-type products are now marketed by several feed and specialty additive companies and are used in multiple aquaculture regions, including the European Union and major Asian production markets such as Thailand, Vietnam, and China. This level of adoption reflects growing confidence in their processing stability and practical utility, although product-specific efficacy should still be verified under target species, diet composition, and production conditions. Because of its relative maturity, MSB provides a useful benchmark for evaluating newer postbiotic and metabolite-delivery technologies, including polymer-coated pellets, nano-emulsions, and hybrid SCFA–exopolysaccharide matrices in seabass and other marine teleost systems [110].

### 5.3. Alginate-Based Encapsulation for Whole-Cell Paraprobiotics

Sodium alginate-based encapsulation is particularly suitable for whole-cell paraprobiotic preparations that require protection during feed processing, gastric passage, and water exposure, followed by release in the intestinal tract. The sodium alginate–CaCl_2_ gelation system typically produces beads ranging from approximately 0.5 to 3 mm in diameter. These beads remain relatively stable under acidic gastric conditions because calcium-mediated crosslinking maintains the integrity of the alginate matrix. At higher intestinal pH, the matrix can swell, erode, or partially dissolve through ion exchange and gradual loss of calcium crosslinks, allowing the encapsulated microbial cells or cell-derived structures to be released closer to the intended site of action [111].

Linh et al. (2026) demonstrated that alginate-based encapsulation of *Lysinibacillus* sp. PWR01 improved its efficacy in Nile tilapia compared with non-encapsulated preparations, supporting the value of this delivery platform for microbial-based feed additives [79]. Although this study focused on probiotic delivery, the same encapsulation principle is directly relevant to heat-killed paraprobiotic formulations, where preservation of whole-cell structure and controlled intestinal release are important. Sihamok et al. (2026) [112] similarly applied algae-derived polysaccharide-based co-encapsulation of *Bacillus* sp. THPS1 for heat-stable delivery in poultry, providing additional evidence that polysaccharide-based encapsulation systems can be scaled for thermally processed feeds. In shrimp, Adilah et al. (2022) showed that alginate–chitosan double encapsulation of *Bacillus subtilis* E20 improved stability during *L. vannamei* feed pelletization and intestinal transit, supporting the feasibility of this platform for aquafeed applications [113]. Together, these findings suggest that alginate-based and alginate–chitosan encapsulation systems may provide practical delivery options for whole-cell paraprobiotics in fish and shrimp, although formulation performance should be validated for each target species, feed process, and culture condition.

### 5.4. Nanoencapsulation and Emerging Delivery Systems

Nanoencapsulation provides additional opportunities to improve the stability, retention, and mucosal delivery of postbiotic and paraprobiotic preparations in aquafeeds. Chitosan nanoparticles, typically ranging from approximately 200 to 500 nm, are particularly attractive because of the mucoadhesive interaction between positively charged chitosan and negatively charged mucin glycoproteins in the teleost intestinal mucus layer. This interaction may prolong contact between the encapsulated bioactive cargo and the gut epithelium, thereby improving local retention and potentially enhancing immune or barrier-related responses [114]. Although direct aquaculture evidence remains limited, Fattahi et al. (2025) demonstrated the use of ZnO–lignin–chitosan nanoparticles for ciprofloxacin delivery against *Pseudomonas aeruginosa* biofilms [115]. This study provides a useful design precedent for adapting chitosan-based nanoparticle systems to deliver bacteriocins, antimicrobial peptides, or other proteinaceous postbiotic components in aquaculture settings.

Outer membrane vesicles (OMVs) and bacterial extracellular vesicles (BEVs) represent another emerging delivery platform. These naturally produced vesicles, generally ranging from 20 to 300 nm, contain microbial lipids, proteins, nucleic acids, and other immunologically active molecules. Because of their nanoscale size and intrinsic microbial composition, OMVs may function both as bioactive postbiotic-like structures and as delivery vehicles without requiring additional encapsulation for some aqueous applications [115]. This property makes them potentially suitable for immersion-based delivery in larviculture, where feed intake is limited or variable. However, their use in aquaculture will require careful evaluation of vesicle composition, dose, safety, stability in water, and species-specific immune responses. Wei et al. (2025) reviewed next-generation probiotics and engineered BEV platforms in biomedical contexts, providing a conceptual framework that may inform future adaptation of OMV/BEV-based delivery systems for aquaculture [21]. At present, these technologies remain promising but exploratory, and direct validation in fish and shrimp feeding or immersion challenge models is needed before they can be considered practical postbiotic delivery tools.

### 5.5. Practical Formulation and Quality-Control Requirements

For commercial translation, formulation claims should be supported by product-level quality-control data rather than by biological performance outcomes alone. At a minimum, future studies should report the production strain and its genome-based safety profile, the inactivation method used and confirmation of sterility, chemical or structural characterization of the active fraction, dose expressed in biologically meaningful units, pellet-processing temperature and pressure, shelf-life stability, water-leaching rate, palatability, and recovery of the active component after feed manufacture. These parameters are especially important for products containing SCFAs, cell-free fractions, bacteriocin-like compounds, and OMVs, because their concentration, stability, and biological activity may change substantially during processing, storage, and immersion before ingestion.

A practical translational framework should also distinguish among products designed for grow-out feeds, hatchery feeds, live-feed enrichment, and short-term therapeutic-support feeding. These production settings differ in pellet size, water exposure time, feeding frequency, developmental stage, immune maturity, and disease risk. Therefore, a formulation that performs well in juvenile tilapia grow-out diets may not be directly applicable to shrimp hatcheries, marine fish larvae, or salmonid smolt feeds without delivery-specific validation. Establishing standardized reporting criteria for formulation properties, processing stability, and target-use conditions will be essential for comparing studies and advancing postbiotic and paraprobiotic products from experimental trials to reliable commercial applications.

### 5.6. Quantification and Standardization of Non-Viable Microbial Preparations

Because postbiotic and paraprobiotic preparations differ substantially in composition, no single quantitative unit is appropriate for all products. Whole-cell preparations should be characterized by the identity of the progenitor microorganism, the cell concentration before inactivation, a post-inactivation measure of total cells or cell equivalents where feasible, and confirmation that deliberate inactivation has occurred. Cell-disrupted or mixed preparations should additionally be standardized using composition-specific markers, such as total protein, peptidoglycan, β-glucan, or other defined cellular components. For preparations containing microbial metabolites, the relevant analytes should be quantified directly. Product standardization should also include batch composition, processing and storage stability, and recovery of the active material after feed manufacture. Thus, standardization requires both quantitative composition and process-level quality control rather than reliance on a single unit such as CFU.

Several complementary methods can be used to quantify non-viable microbial preparations. For whole-cell products, the starting culture concentration may be recorded before inactivation, while total cell abundance after treatment can be estimated by flow cytometry or direct microscopic counting and reported as cell equivalents. Dry biomass and total protein can provide additional batch-level measures. Molecular approaches, including quantitative or digital PCR, may support biomass characterization, although detection of microbial DNA does not by itself distinguish viable from non-viable cells. When specific structural components are relevant to product activity, peptidoglycan, β-glucan, EPS, or protein content can be quantified using appropriate biochemical or immunochemical assays. SCFAs and other defined metabolites can be measured by chromatographic methods such as HPLC or GC. Extracellular vesicle preparations should additionally report particle concentration and size distribution. Culture-based CFU measurements after inactivation are most useful for assessing residual viable cells rather than quantifying the total non-viable preparation.

## 6. Challenges, Future Perspectives, and Research Priorities

**Figure 6:**
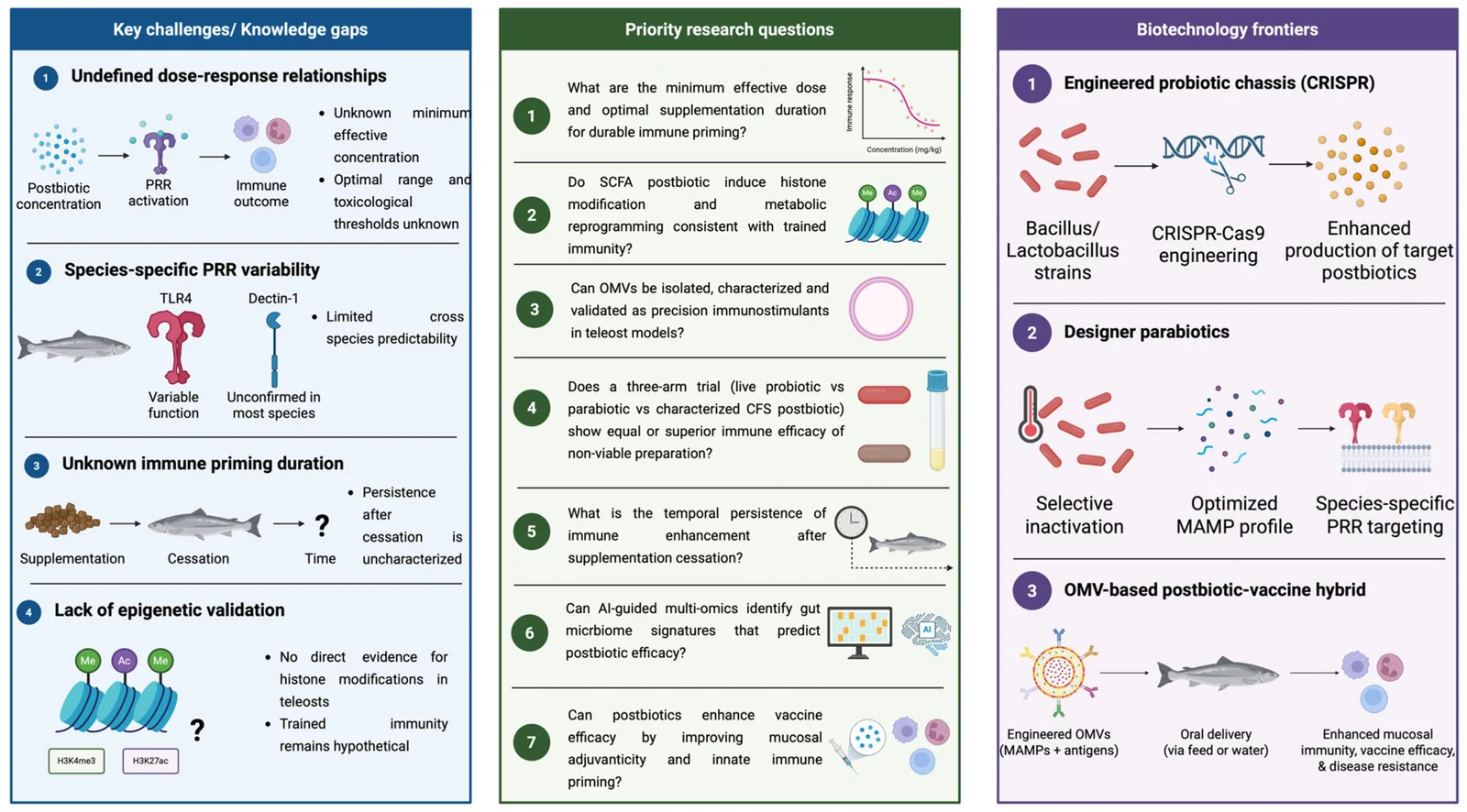
Future research and commercial translation roadmap for aquaculture postbiotics and paraprobiotics. This evidence-to-translation pipeline outlines the main steps required to move candidate products from discovery to commercial applications. It highlights that promising biological responses alone are insufficient to support commercial claims. Candidate products should be clearly defined by strain identity, production method, inactivation or fractionation procedure, and analytical composition, and then evaluated through dose–response studies, feed-processing stability tests, safety assessments, controlled efficacy trials, and validation under farm-relevant conditions.

Although postbiotics and paraprobiotics show promise in aquaculture, their practical application remains limited by gaps in product definition, mechanism of action, formulation stability, and farm-level validation. Moving these products toward commercial use will require more than improved growth or survival in controlled trials. Future studies should clearly define product composition, explain the likely mechanisms involved, evaluate stability during feed production and water exposure, and confirm whether the observed benefits can be reproduced under realistic farming conditions (Figure 6).

### 6.1. Scientific and Technical Knowledge Gaps

Despite the growing evidence base, several fundamental limitations continue to hinder the mechanistic characterization and predictive application of postbiotics and paraprobiotics in aquaculture (Table 4). Dose–response relationships remain poorly defined, as most published studies use only one or two inclusion levels and rarely quantify the relationship among intestinal postbiotic concentration, pattern-recognition receptor activation, and the magnitude of immune or protective outcomes. As a result, minimum effective concentrations, optimal dose ranges, and potential toxicological thresholds cannot yet be established for most preparations. Species-specific differences in pattern-recognition receptor repertoires also create uncertainty when findings are extrapolated across aquaculture taxa. This issue is particularly relevant for variable TLR4 functionality and the still-incompletely characterized Dectin-1-like recognition systems in many commercially important species. In addition, the duration of immune priming after supplementation withdrawal remains largely uncharacterized in fish and crustaceans, limiting the design of practical feeding schedules for commercial use. Direct epigenetic characterization of postbiotic-treated fish immune cells is also scarce. Therefore, although the trained-immunity hypothesis for SCFA-containing preparations is mechanistically plausible, it remains insufficiently validated in aquaculture species. Addressing these gaps will require studies that combine controlled dose–response designs, receptor-level assays, longitudinal immune monitoring, chromatin-level analyses, and pathogen challenge models across representative fish and shrimp species.

### 6.2. Priority Research Questions

Several priority research questions emerge from this synthesis. Addressing these questions would help move the field beyond empirically reported outcomes, limited mechanistic grounding, and poorly defined dose–response relationships toward evidence-based formulation standards suitable for regulatory evaluation and commercial application. A key need is to define the minimum effective doses and optimal supplementation durations required for each postbiotic structural category to induce durable innate immune priming in commercially important aquaculture species under realistic production conditions. It is also important to determine whether SCFA-containing preparations can induce histone modification marks, such as H3K4me3 and H3K27ac, together with metabolic reprogramming consistent with trained immunity in teleost innate immune cells. Another priority is to isolate and characterize OMVs from aquaculture-relevant probiotic Bacillus strains using approaches such as transmission electron microscopy and nanoparticle tracking analysis, followed by validation of their immunostimulatory effects in teleost challenge models.

Well-controlled comparative studies are also needed to distinguish the relative contributions of viable cells, inactivated whole cells, and secreted microbial products. A three-arm trial comparing a live probiotic, a heat-killed paraprobiotic, and a characterized cell-free supernatant or metabolite-rich fraction from the same strain, tested at equivalent bioactive doses in the same host species and challenge model, would provide especially valuable mechanistic insight. Additional research should determine how long measurable immune enhancement persists after dietary supplementation is withdrawn and whether this persistence differs among structural categories. Multi-omics approaches, including metagenomics, host transcriptomics, and metabolomics, may also help identify gut microbiome signatures that predict postbiotic efficacy in individual fish. Finally, future studies should evaluate whether postbiotic supplementation can improve the performance of existing aquaculture vaccines by enhancing mucosal adjuvanticity and innate immune priming at the time of vaccination.

### 6.3. Biotechnological Frontiers

Several emerging biotechnological approaches could expand the design space for postbiotic and paraprobiotic development in aquaculture. In the near term, CRISPR-based metabolic engineering of probiotic Bacillus chassis strains may provide a defined route to enhance the production of specific postbiotic outputs, including butyrate through amplification of the butyryl-CoA pathway, lipopeptides such as iturin A and surfactin through modification of PKS/NRPS biosynthetic gene clusters, and selected exopolysaccharide types [126]. Compared with conventional strain selection, this approach could improve product consistency and increase the yield of targeted bioactive compounds. However, its practical application will require careful evaluation of strain safety, genetic stability, regulatory acceptance, and containment, particularly when engineered organisms are used only as production platforms for non-viable preparations.

Designer paraprobiotics represent another promising direction. These preparations could be produced using selective inactivation methods that preserve or enrich specific microbial-associated molecular pattern profiles. This would move paraprobiotic development beyond empirical heat treatment toward more rational immunobiotic manufacturing, in which product composition is analytically confirmed and matched to the pattern-recognition receptor repertoire of the target aquaculture species. Such an approach may allow more predictable immune modulation, especially when specific MAMPs are linked to desirable outcomes such as barrier reinforcement, controlled inflammatory priming, or improved resistance to bacterial challenge.

OMVs and BEVs engineering may represent one of the most innovative frontiers in this field. Surface-modified vesicles derived from well-characterized probiotic *Bacillus* or *Lactobacillus* strains could, in principle, be designed to deliver both trained-immunity-inducing MAMPs and pathogen-specific immunogenic antigens, such as *Aeromonas* OmpA epitopes or Streptococcus M-protein fragments. This concept would create a next-generation postbiotic–vaccine hybrid nanoparticle that combines microbial immune stimulation with antigen-specific targeting. Such platforms could be especially valuable for oral, immersion, or live-feed delivery, where conventional injectable vaccines are impractical for small fish, larvae, or some shrimp production stages. The expanding literature on BEVs engineering in mammalian therapeutics provides a useful conceptual and technical foundation for this aquaculture application, although direct validation in fish and shrimp remains at an early stage [21].

### 6.4. Minimum Reporting Standards for Future Aquaculture Postbiotic Studies

To improve reproducibility and allow meaningful comparison among studies, future research on aquaculture postbiotics and paraprobiotics should report a consistent set of core experimental and product-level details. These should include full taxonomic identification of the source strain, with deposit or accession information where available; genome-based screening for antimicrobial-resistance genes, virulence factors, mobile genetic elements, and toxin-related loci when relevant; and a clear description of the inactivation or fractionation method used. For paraprobiotic preparations, evidence confirming complete loss of viability should be provided. Studies should also include analytical characterization of the product, such as cell equivalents, total protein content, SCFA concentration, EPS content, OMV size distribution, or other bioactive markers appropriate to the preparation. In addition, stability during feed processing and water exposure should be evaluated, particularly for products intended for pelleted aquafeeds or immersion-based delivery.

Experimental design should be reported with sufficient detail to support interpretation and replication. This includes tank-level replication and randomization, appropriate negative, vehicle, and positive-control diets, dose–response assessment, and sampling after withdrawal of supplementation. Relevant biological outcomes should also be measured, including growth performance, feed conversion ratio, histology, immune gene expression, microbiome composition, pathogen load, and survival following standardized challenge. In feeding trials, the tank, cage, or aquarium should generally be treated as the experimental unit rather than the individual fish or shrimp, unless animals are housed independently. Challenge trials should report the pathogen dose, exposure route, confirmation of virulence, mortality criteria, and pathogen re-isolation or molecular confirmation. Without these details, reported improvements in survival, immune responses, or disease resistance remain difficult to interpret and may not be reproducible across laboratories or production systems.

### 6.5. Limitations of the Current Evidence Base

Several limitations in the current evidence base should be acknowledged. Terminology remains inconsistent, with terms such as postbiotic, paraprobiotic, metabolite, and cell-free supernatant often used interchangeably, which complicates comparison among studies. Many preparations are also insufficiently characterized, making it difficult to determine which microbial components or metabolites are responsible for the reported biological effects. In addition, most studies evaluate only one or two dietary inclusion levels, limiting conclusions about minimum effective doses, optimal supplementation ranges, and upper safety thresholds. Laboratory challenge models also have important constraints, as they often use high pathogen doses and may not fully reflect the combined effects of stress, co-infection, variable water quality, and fluctuating environmental conditions encountered in commercial farms. Another limitation is that upregulation of immune-related genes alone does not confirm pathway activation, trained-immunity-like responses, or improved disease resistance. Findings from a single host species, microbial strain, preparation type, or delivery matrix therefore should not be broadly generalized without validation in the intended production system. Addressing these limitations will be essential for strengthening mechanistic interpretation and improving the translational value of future postbiotic and paraprobiotic studies in aquaculture.

### 6.6. Practical Applications and Broader Significance

Near-term application is most feasible for products with demonstrated stability, safety, and measurable farm-level benefits. Examples include protected butyrate or SCFA-derived additives for plant-protein-rich diets, yeast cell-wall or β-glucan products for immune support in shrimp and salmonids, heat-killed *Bacillus* or *Lactobacillus* preparations for high-temperature pelleted feeds, and short-term hatchery or nursery supplementation during predictable periods of disease risk. In these settings, postbiotic and paraprobiotic products should be considered components of integrated health management. They may complement biosecurity, vaccination where available, water-quality management, selective breeding, diagnostics, and responsible therapeutic use, rather than replace these measures entirely. Unlike live probiotics, which are routinely used in some shrimp biofloc and water-management systems, comparable routine farm-scale use of postbiotics and paraprobiotics has not yet been well documented. Current applications are mainly feed-based, and evidence for routine water-column applications comparable to probiotic use in biofloc systems remains limited.

Commercial translation is currently most advanced for several non-viable microbial and microbial-derived feed additives. Feed LP20 contains heat-killed *L. plantarum* L-137 and has been evaluated as a dietary ingredient in salmonids [78]. MacroGard is a *Saccharomyces cerevisiae*-derived β-1,3/1,6-glucan product with a long history of use as an immunomodulatory feed ingredient in aquatic animals [82,83]. Protected butyrate formulations are also commercially available for incorporation into functional feeds. These examples illustrate that some non-viable microbial preparations and microbial-derived products have already progressed beyond experimental development. However, commercial availability should not be equated with classification as an ISAPP-defined postbiotic. Purified β-glucan and butyrate, when administered independently of an inanimate microbial preparation, should be considered microbial-derived functional feed additives rather than postbiotics. In addition, product availability, formulation, authorized claims, and regulatory classification vary among regions.

Practical adoption will further depend on production cost, manufacturing scalability, and the ability to maintain product activity during feed manufacture and storage. Heat inactivation followed by drying or direct feed incorporation is relatively straightforward to scale, whereas irradiation, high-pressure processing, vesicle purification, and nanoencapsulation may require specialized equipment and additional quality-control steps. Regulatory requirements are also jurisdiction-dependent and may differ according to whether a product is marketed as a feed ingredient, functional additive, immunomodulator, or product carrying disease-prevention or therapeutic claims.

Compared with live probiotics, non-viable preparations do not require maintenance of cell viability during feed processing or storage and may reduce concerns associated with persistence or transfer of antimicrobial-resistance determinants from replicating supplemented microorganisms. Synbiotics combine live probiotics with substrates intended to support their activity and may provide broader microbiome modulation, but their performance still depends partly on probiotic viability and persistence. In contrast, postbiotics and paraprobiotics offer greater processing stability and product consistency, although they cannot proliferate or continuously produce metabolites after administration. These approaches should therefore be considered complementary rather than directly interchangeable, with their suitability depending on the species, production system, and intended application. Overall, practical suitability should be evaluated case by case according to product stability, effective dose, delivery route, manufacturing cost, regulatory status, and evidence of efficacy under farm conditions. At present, feed-based applications appear more readily transferable than routine water-column use, but wider adoption will require stronger validation under commercial production conditions.

More broadly, this field offers an opportunity to move microbiome-based aquaculture health management from empirically selected live probiotics toward more defined, stable, and quality-controlled microbial products. With stronger evidence from dose–response studies, safety assessment, mechanistic validation, and farm-scale trials, postbiotics and paraprobiotics could help reduce antibiotic reliance, improve resilience under intensive farming conditions, support the use of sustainable plant-based feeds, and provide more predictable products for producers and regulators.

## 7. Conclusions

This review summarizes current evidence on postbiotics and paraprobiotics in aquaculture, with emphasis on structural classification, immune-recognition pathways, species-specific responses, feed-delivery challenges, and remaining knowledge gaps. Available studies suggest that heat-killed microbial preparations, SCFA-containing formulations, and yeast cell-wall products can support innate immunity, intestinal barrier function, and disease resistance under controlled experimental conditions, although the strength of evidence varies across species, product types, and delivery systems. The proposed role of SCFA-related preparations in trained-immunity-like responses is biologically plausible, but direct chromatin-level evidence in commercially important aquaculture species remains limited. Future progress will require clearer dose–response studies, better product characterization, feed-processing stability data, and farm-scale validation. With these requirements addressed, postbiotics and paraprobiotics may become useful components of integrated health-management strategies aimed at improving resilience and reducing antibiotic reliance in intensive aquaculture.

## Figures and Tables

**Table 1:** Structural classification of non-viable microbial preparations and related products: primary characteristics and evidence base in aquaculture.

Category	Key Producing Organisms	Main Bioactive Components	Host Target(s)/Mechanisms	Stability in Aquafeed	Evidence Level in Aquaculture	References
Inactivated cellular preparations	*Bacillus* spp., *Lactiplantibacillus* spp., *Pediococcus* spp.	Inactivated whole cells containing multiple cell-wall and intracellular components	Multivalent PRR engagement; TLR-, NOD-, and other innate immune pathways depending on preparation	Generally higher than live probiotics; depends on inactivation and formulation method	Growing; several feeding and challenge studies in fish and shrimp	[9,16]
Cell-wall and structural components	*Bacillus* spp.; *Saccharomyces cerevisiae*; selected Gram-negative bacteria	PGN, LTA, MDP, β-1,3/1,6-glucan, LPS-associated structures	NOD1/2; TLR2-associated signaling; CLR-like and CR3 pathways; responses to Gram-negative components are species dependent	Generally high, particularly for yeast cell-wall preparations	Strong for yeast β-glucan; increasing evidence for bacterial PGN/LTA preparations	[17,18,19,20]
Microbial polysaccharides and extracellular polymers	*Bacillus* spp., *Lactiplantibacillus* spp., *Leuconostoc* spp., *Pediococcus* spp.	EPS, levan, kefiran, dextran, and related polysaccharides	Modulation of mucosal immunity, barrier function, and lectin-associated pathways	Generally good; many polysaccharides are relatively heat stable	Moderate; effects vary with polymer structure and host species	[17,18]
Extracellular vesicles and membrane-derived structures	Gram-negative probiotic or commensal bacteria; selected Gram-positive bacteria	OMVs/BEVs containing lipids, proteins, PGN fragments, nucleic acids, and other microbial components	Multivalent innate immune signaling through membrane and intracellular PRRs	Relatively stable because of the lipid-bilayer structure, but formulation-dependent	Emerging; direct aquaculture evidence remains limited	[21]
Microbial proteins and peptides	*B. subtilis*, *Lactiplantibacillus plantarum*, *Pediococcus acidilactici*, and other microbial sources	Bacteriocins, antimicrobial peptides, lipopeptides, HSPs, and other functional proteins	Direct antimicrobial activity; selected components may also influence PRR-associated immune signaling	Variable; many proteins and peptides are sensitive to heat or proteolysis	Moderate for antimicrobial activity; direct in vivo aquaculture evidence remains variable	[8,16]
Microbial metabolites and fermentation-associated products	*Clostridium butyricum*, *Bifidobacterium* spp., *Lactiplantibacillus* spp., *Bacillus* spp., and other fermentative microorganisms	Acetate, propionate, butyrate, lactic acid, citric acid, B vitamins, and other metabolites	HDAC inhibition and FFAR2/3 signaling for selected SCFAs; pH-mediated pathogen inhibition; metabolic and barrier-related effects	Variable; organic-acid salts are generally more stable, whereas some vitamins and volatile compounds may require protection	Strongest for butyrate in selected finfish; evidence varies for other metabolites	[8,22,23,24,25,26,27]

PRR, pattern recognition receptor; PGN, peptidoglycan; LTA, lipoteichoic acid; MDP, muramyl dipeptide; EPS, exopolysaccharide; OMV, outer membrane vesicle; BEV, bacterial extracellular vesicle; HSP, heat-shock protein; HDAC, histone deacetylase; FFAR, free fatty acid receptor; CLR, C-type lectin receptor; CR3, complement receptor 3.

**Table 3:** Summary of selected in vivo postbiotic and paraprobiotic evidence in key aquaculture species.

Inactivation/ Product	Source Strain/Product	Aquatic Species	Dose	Duration	Challenge Pathogen	Main Outcomes	Reference
Heat-killed lactic acid bacteria	*Lactiplantibacillus plantarum* L-137 (Feed LP20)	Atlantic salmon pre-smolts; rainbow trout cell-line model	20, 100, 500 mg/kg feed	61 days	NR	Improved intestinal barrier function; increased IL-1β and plasma IgM; decreased Anxa1; broad intestinal gene modulation without growth impairment	[78]
Heat-killed lactic acid bacteria	*L. plantarum* (heat-killed)	Giant freshwater prawn (*Macrobrachium rosenbergii*)	NR	NR	*A. hydrophila*	No significant growth effect; increased total haemocyte count, phenoloxidase activity, respiratory burst, and bacterial clearance; reduced mortality after *A. hydrophila* challenge	[93]
Heat-killed/postbiotic LAB	*Pediococcus pentosaceus* PP4012 (heat-killed postbiotic)	Pacific white shrimp (*Litopenaeus vannamei*)	10^5^ and 10^6^ cell equivalents/g feed	56 days	*V. parahaemolyticus*	Increased final weight, SGR, and feed utilization; reduced mortality after *V. parahaemolyticus* challenge; increased lysozyme and phagocytic activity; altered gut microbiota	[89]
Heat-killed *Bacillus* paraprobiotic	*B. siamensis* LF4 (HKBS)	Spotted seabass (*Lateolabrax maculatus*)	NR	NR	NR	Mitigated high-soybean-meal-induced growth suppression and liver/intestinal injury; improved immune indices; reshaped intestinal microbiota	[94]
Heat-killed *Bacillus* paraprobiotic	*B. coagulans* (heat-killed)	Pacific white shrimp (*Litopenaeus vannamei*)	NR	90 days	*V. parahaemolyticus*	After 90 days, improved growth and FCR; increased survival after *V. parahaemolyticus* challenge; increased SOD, catalase, nitric oxide, haemocyte, and PO responses	[95]
Heat-killed *Bacillus* preparation	*B. amyloliquefaciens* FPTB16 (heat-inactivated)	Catla (*Catla catla*)	NR	NR	NR	Increased oxygen radical production, lysozyme, total protein, myeloperoxidase, and alkaline phosphatase; upregulated IL-1β, TNF-α, C3, and iNOS	[96]
Formalin-inactivated *Bacillus* preparation	*B. amyloliquefaciens* COFCAU_P1 (heat- and formalin-inactivated; formalin used in vivo)	Rohu (*Labeo rohita*)	10^8^ cells/g feed showed the strongest response	NR	*A. hydrophila*	In vitro, both heat- and formalin-inactivated preparations increased superoxide, MPO, NO, leukocyte proliferation, and IL-1β/IFN-γ expression; in vivo, formalin-inactivated diets improved innate immunity and protection against *A. hydrophila*, with the best response at 10^8^ cells g^−1^	[97]
Sonication-killed cell-free extract	*Clostridium butyricum* CBG01 (sonication-killed extract, heat-killed whole cells, and live cells)	Pacific white shrimp (*Litopenaeus vannamei*)	NR	NR	*V. parahaemolyticus*	All forms improved growth and immune enzyme activities; the sonication-killed extract provided the strongest resistance to *V. parahaemolyticus*, while heat-killed cells also enhanced several immune genes	[98]
Paraprobiotic and postbiotic mixture	*B. subtilis*-derived paraprobiotic/postbiotic preparation	Asian seabass (*Lates calcarifer*)	NR	NR	*S. iniae*	Increased growth, serum immune parameters, antioxidant status, TNF-α, IL-6, IL-1β, and PRR-related genes (NLRC3, TLR22, MDA5); improved survival after *S. iniae* challenge	[12]

NR, not reported.

**Table 4:** Key knowledge gaps in aquaculture postbiotic/paraprobiotic research and proposed methodological approaches.

Knowledge Gap	Current Status/Missing Information	Proposed Methodological Approaches	References
Trained immunity and epigenetics in fish	Trained immunity has been described in teleosts, including candidate inducers, effector cells, and mechanisms, but activating chromatin marks in fish macrophages remain poorly mapped. Mammalian studies provide well-defined histone and epigenomic signatures of trained monocytes/macrophages.	Adapt ChIP-seq and ATAC-seq workflows to head-kidney macrophages to map activating histone marks and open chromatin after postbiotic exposure, using mammalian trained-immunity pipelines as templates.	[116,117,118]
SCFA dose–response and kinetics	Dietary SCFAs, including acetate, propionate, and butyrate, can improve antioxidant enzymes, mucosal and humoral immunity, gut-barrier genes, microbiota, and disease resistance in crucian carp and zebrafish, but most studies use a single high dose and limited time points.	Conduct systematic dose-titration, time-course, and withdrawal studies, with concurrent measurement of gut SCFA concentrations and immune outputs. Include lower butyrate ranges relevant to trained-immunity studies where appropriate.	[119,120,121]
PRR targets of postbiotics in fish	Teleost TLR, RLR, and NLR repertoires are well catalogued, but ligands and adaptor usage remain unresolved for many receptors. Some ray-finned fishes also lack canonical mammalian LPS co-receptors and show divergent TLR4 pathways.	Use comparative PRR genomics and transcriptomics across species, together with in vitro reporter assays, to define which TLRs, NLRs, and CLRs respond to specific postbiotic structures, including β-glucans, peptidoglycan fragments, and OMVs.	[53,122,123]
Bacterial EVs/OMVs as postbiotics	Gram-negative OMVs and Gram-positive EVs are recognized as potent immunomodulators and vaccine/adjuvant platforms in mammals. Fish evidence is emerging, including *Bacillus subtilis* EV activation of pro-inflammatory and B-cell responses in trout leukocytes, while probiotic *E. coli* OMVs modulate macrophage cytokines and antibacterial activity in mammalian models.	Standardize OMV/EV isolation and characterization, including size distribution and cargo profiling. Conduct factorial in vivo trials comparing live probiotics, purified EVs, and killed cells, followed by long-term safety and efficacy testing in aquaculture species.	[124,125]
Distinguishing probiotics, paraprobiotics, and postbiotics in fish	Recent reviews distinguish live probiotics, inactivated paraprobiotics, and metabolite- or fraction-based postbiotics, but many experimental studies still use these terms inconsistently.	Use three-arm trials that compare the live strain, heat-killed cells, and secreted fractions or SCFA-rich preparations from the same source strain, with shared endpoints for growth, microbiota, immune responses, and disease outcomes.	[8,13]
Multi-omics integration of gut microbiome-postbiotic-immunity links	Functional feeds, including SCFAs and other microbiome-associated metabolites, are recognized as important modulators of the fish gut microbiome and immune system. GC-MS, NMR, and metagenomics are increasingly used to profile metabolites and microbiota.	Design integrated trials that combine 16S/metagenomics, targeted SCFA metabolomics, host transcriptomics, and immune phenotyping after postbiotic/paraprobiotic feeding to link specific metabolites and taxa with immune outcomes.	[120,121]
Long-term safety, ecology, and implementation	Reviews highlight regulatory, biosafety, horizontal gene transfer, and long-term ecological concerns associated with functional feeds and microbiome modulation in aquaculture, as well as the need for sustainable implementation.	Conduct long-duration field trials that monitor performance, pathogen dynamics, resistance patterns, and environmental microbiomes, and develop regulatory-aligned safety dossiers for postbiotic and paraprobiotic products.	[8,121]

## Data Availability

This review article is based on previously published studies. No new datasets were generated or analyzed during the current work.
